# A Universal Approach Using Water‐Soluble Templates for Meso‐ and Macro‐Porous Organic Polymers

**DOI:** 10.1002/advs.202508489

**Published:** 2025-07-22

**Authors:** Yusuke Asakura, Steven Adiwijaya, Shunya Yoshino, Hideki Kato, Yusuke Yamauchi

**Affiliations:** ^1^ Department of Materials Process Engineering Graduate School of Engineering Nagoya University Furo‐cho, Chikusa‐ku Nagoya Aichi 464‐8603 Japan; ^2^ Institute of Multidisciplinary Research for Advanced Materials (IMRAM) Tohoku University Sendai Miyagi 980‐8577 Japan; ^3^ Australian Institute for Bioengineering and Nanotechnology (AIBN) The University of Queensland Brisbane Queensland 4072 Australia

**Keywords:** meso‐ and macro‐pores, perovskite fluorides, porous organic polymers, Schieff reactions, water‐soluble templates

## Abstract

Porous functional organic polymers have attracted significant interest due to their diverse applications in adsorption/separation, electrocatalysis, photocatalysis, photosensing, and electronics. In these applications, performance depends on interactions between guest molecules and polymer frameworks. Consequently, the introduction of mesopores (2–50 nm) and macropores (50–300 nm) can significantly enhance functionality by simultaneously increasing the exposed active surface area and facilitating molecular diffusion within the polymer matrix. However, a wide range of porous polymers achievable through previous approaches, including surfactant and polystyrene templating, has been severely limited to a few less functional polymers due to the typically aqueous environment used during templating reaction and the need for template removal after polymerization. In this work, a universal approach is demonstrated that utilizes water‐soluble templates to synthesize functional meso‐ and macro‐porous organic polymers via solid‐state polymerization of aldehydes and amines (Schiff reaction), using perovskite metal fluorides (K*M*F_3_) as sacrificial templates. By precisely tuning the particle sizes of metal fluoride templates, accurate control over pore sizes across the mesoscopic and macroscopic scales is achieved. A variety of aldehyde‐amine combinations yield semiconductive meso‐ and macro‐porous organic polymers. This versatile synthetic strategy is broadly applicable to a wide range of polymer systems, enabling simultaneous enhancement of surface area and molecular diffusion, thereby optimizing functional performance.

## Graphical abstract

A versatile solid‐state strategy using water‐soluble perovskite fluorides enables the synthesis of meso‐ and macro‐porous covalent organic polymers. The pore size is tunable by the template particle size, and the method overcomes the compositional limitations of conventional templating approaches, offering broad applicability in porous polymer design.

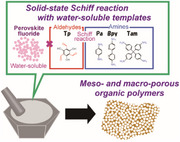

## 1 Introduction

Porous organic polymers have garnered significant attention due to their diverse applications^[^ ^1^, ^2^, ^3^, ^4^ ^]^ in adsorption/separation,^[^ ^5^, ^6^ ^]^ catalysts,^[^ ^7^ ^]^ electrocatalysis,^[^ ^8^, ^9^ ^]^ photocatalysis,^[^ ^10^, ^11^, ^12^ ^]^ and sensors.^[^ ^13^, ^14^ ^]^ The integration of nanopores into polymers enables unique functionalities by synergistically combining the intrinsic properties of the polymer backbone with the benefits of nanoporosity. Porous structures not only enhance the accessible surface area but also improve the diffusion of guest molecules within the materials. The functional role of pores depends critically on their size: macropores (>50 nm) facilitate reagent diffusion but contribute minimally to surface area, while micropores (<2 nm) provide a large surface area but hinder reagent diffusion due to their confined dimensions. Mesopores (2–50 nm) offer an optimal balance, simultaneously increasing surface area and facilitating molecular transport.^[^ ^15^, ^16^, ^17^, ^18^ ^]^ Consequently, the incorporation of mesopores into organic polymers is expected to enhance properties that rely on guest molecule interactions. Although several studies have successfully synthesized mesoporous organic polymers,^[^ ^19^, ^20^, ^21^, ^22^ ^]^ the reported methods lack universal applicability across different polymer systems.

Among various functional organic polymers, those synthesized via Schiff coupling reactions between multifunctional amines and aldehydes have been extensively studied.^[^ ^23^, ^24^ ^]^ However, the introduction of mesopores (10–50 nm) or macropores (50–300 nm) in these polymers remains unexplored. Schiff coupling reactions conducted under solvothermal conditions typically yield crystalline microporous polymers with high specific surface areas (>500 m^2^ g^−1^).^[^ ^23^, ^24^ ^]^ While these microporous structures have been credited with enhancing performance in separation, catalysis, and sensing applications,^[^ ^25^ ^]^ their confined pore size severely limits reagent diffusion, restricting their full functional potential. Thus, precise structural control in organic polymers synthesized via Schiff reactions is essential to achieve both high surface area and efficient molecular transport.

Conventional mesopore‐forming strategies are not directly applicable to Schiff coupling‐based polymer synthesis. Typically, mesoporous materials, such as ceramics (e.g., SiO_2_, TiO_2_),^[^ ^26^ ^]^ metal‐organic frameworks,^[^ ^27^, ^28^, ^29^ ^]^ and organic polymers,^[^ ^20^, ^21^, ^22^ ^]^ are synthesized using surfactant micelles as soft templates. However, this method generally requires water‐soluble precursors capable of interacting with the hydrophilic surfaces of micelles, rendering it unsuitable for the relatively hydrophobic monomers used in Schiff reactions. Alternative approaches, such as employing polystyrene (PS) bead templates, have been utilized to create larger pores in several solids, including silica,^[^ ^30^ ^]^ the other oxides,^[^ ^31^ ^]^ metal‐organic frameworks,^[^ ^32^ ^]^ and covalent organic frameworks.^[^ ^33^ ^]^ However, since PS beads are typically available in sizes exceeding 100 nm, they are unsuitable for generating mesopores (<50 nm). Also, silica nanoparticles are commonly used as hard templates for synthesizing mesoporous materials. However, their removal typically requires harsh conditions, such as treatment with NaOH^[^ ^34^ ^]^ or HF^[^ ^35^ ^]^ aqueous solution, which can limit the compatibility with certain functional materials. Consequently, no established methodology exists for incorporating mesopores into Schiff‐based organic polymers, limiting their applicability in functional materials.

**Scheme 1:**
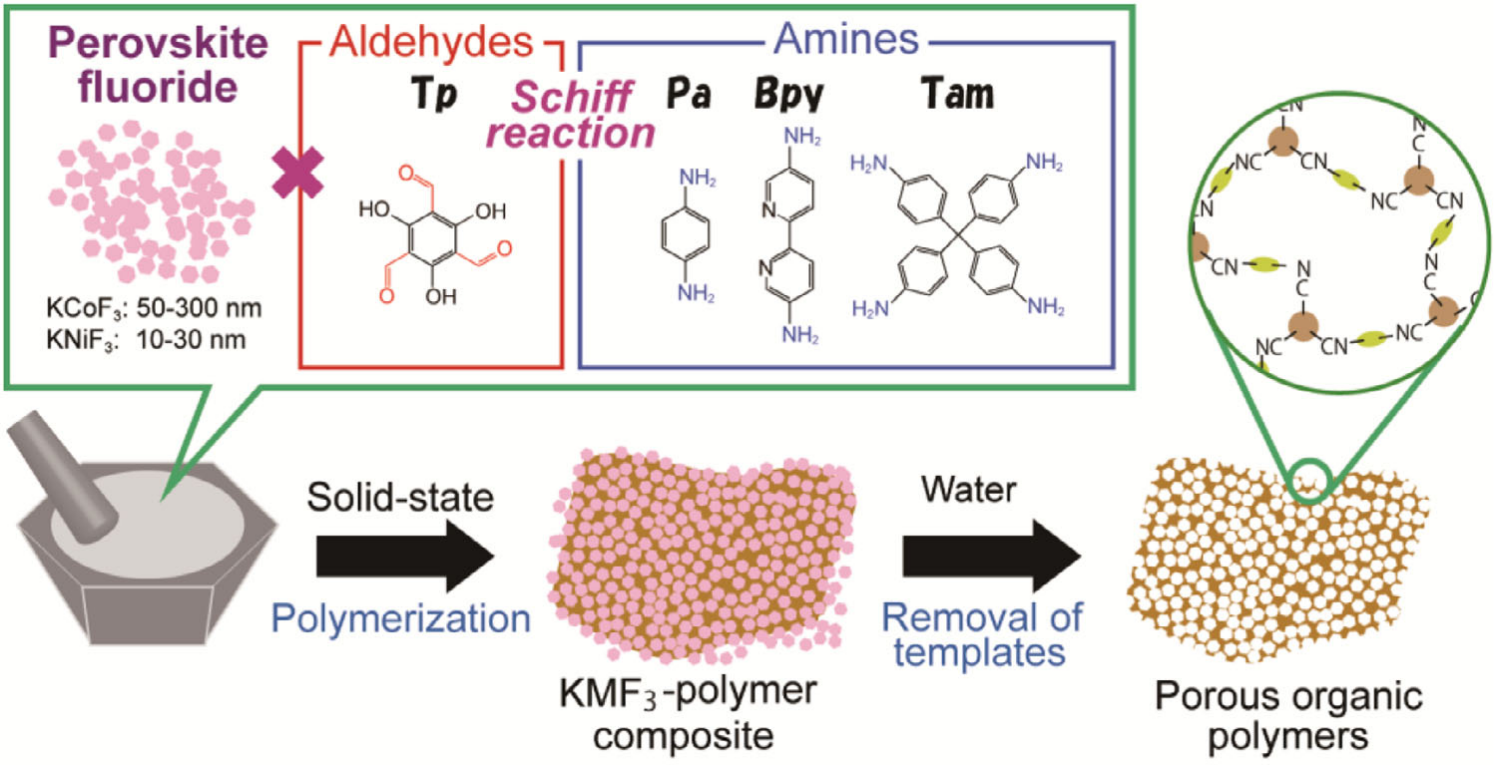
Formation of porous organic polymers by using perovskite fluorides as a water‐soluble template.

In this study, we report a strategy for synthesizing meso‐ and macro‐porous organic polymers via Schiff reactions using water‐soluble perovskite fluorides (K*M*F_3_, *M* = Co, Ni) as sacrificial templates. K*M*F_3_ nanoparticles are readily synthesized via solvothermal methods with tunable sizes and dissolve easily in water. These characteristics enable facile removal of the template under mild conditions and allow precise control over pore size in the resulting polymers by adjusting the particle size of K*M*F_3_. We select mixing the polymer precursors (amines and aldehydes) with a large quantity of K*M*F_3_ nanoparticles in the solid state at room temperature for the polymerization reaction because reactions between amines and aldehydes proceed by solid‐state reaction.^[^ ^36^ ^]^ The polymerization proceeds within the voids between the nanoparticles, eliminating the need for direct strong interaction between the template and monomers. As the Schiff reaction progresses, semiconductive polymers form around the K*M*F_3_ nanoparticles. Subsequent template removal results in meso‐ and macro‐porous polymers with pore sizes dictated by the dimensions of the K*M*F_3_ nanoparticles (**Scheme** **1**). To assess the versatility of this approach, we synthesize meso‐ or macro‐porous semiconductive polymers using various amine‐aldehyde combinations and evaluate their properties. Furthermore, we investigate the photocatalytic hydrogen evolution activity^[^ ^37^ ^]^ of the obtained meso‐ and macro‐porous semiconductive polymers, demonstrating the functional significance of controlled porosity in enhancing photocatalytic performance.

## 2 Results and Discussion

For Schiff reaction‐based polymerization, 2,4,6‐triformylphloroglucinol (Tp) was used as the aldehyde precursor, while p‐phenylenediamine (Pa), [2,2’‐bipyridine]‐5,5’‐diamine (Bpy), tetrakis(4‐aminophenyl)methane (Tam), and 2,6‐diaminoanthraquinone (Dq) were selected as amines. Polymerization was carried out under solid‐state conditions at room temperature in the presence of a large excess of K*M*F_3_ (*M* = Ni, Co) particles. The resulting samples were designated as Tp*Xxx*‐K*M*F_3_, where *Xxx* denotes the amine used (Pa, Bpy, Dq, or Tam) and *M* represents the metal species in the perovskite fluoride (Ni or Co). The particle sizes of KNiF_3_ and KCoF_3_ are ≈10–30 nm and 50–300 nm, respectively, and the porous polymers synthesized using these as templates exhibit corresponding mesopore and macropore sizes.

**Figure 1:**
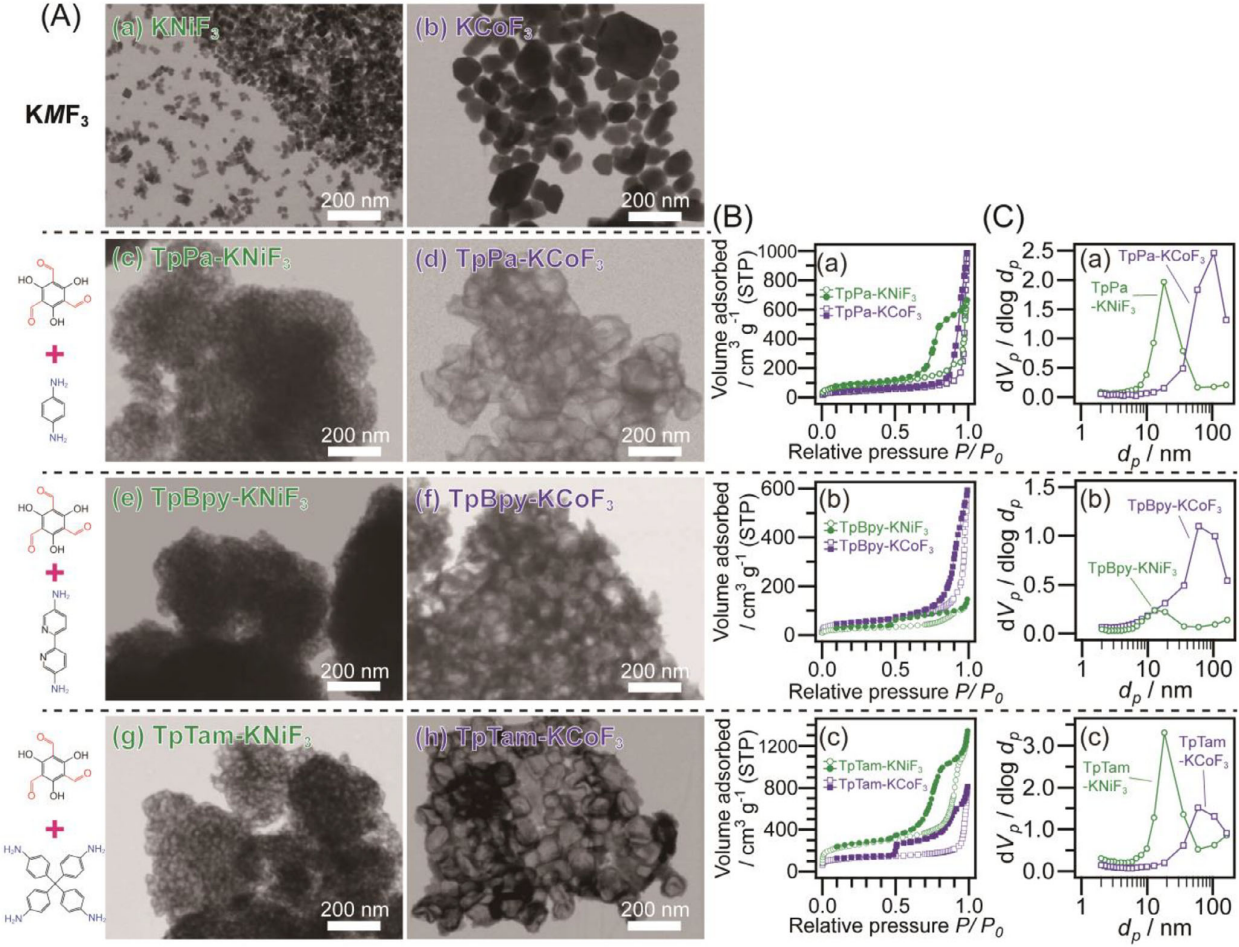
A) STEM images of (a) KNiF_3_, (b) KCoF_3_, (c) TpPa‐KNiF_3_, (d) TpPa‐KCoF_3_, (e) TpBpy‐KNiF_3_, (f) TpBpy‐KCoF_3_, (g) TpTam‐KNiF_3_, and (h) TpTam‐KCoF_3_. B) N_2_ adsorption/desorption isotherms and C) BJH pore size distributions of (a) TpPa‐KNiF_3_, and TpPa‐KCoF_3_, (b) TpBpy‐KNiF_3_, and TpBpy‐KCoF_3_, and (c) TpTam‐KNiF_3_, and (f) TpTam‐KCoF_3_.

The scanning transmission electron microscopy (STEM) images of KNiF_3_, KCoF_3_, and the Tp*Xxx*‐K*M*F_3_ series, along with the N_2_ adsorption/desorption measurements and BJH pore size distributions of the Tp*Xxx*‐K*M*F_3_ series, are summarized in **Figure** **1**. The particle sizes of KNiF_3_ and KCoF_3_ are 10–30 nm and 50–300 nm, respectively (Figure 1A‐a,b), and both exhibit single‐phase structures, as confirmed by XRD patterns (Figure S1, Supporting Information). The TpPa‐KNiF_3_, TpBpy‐KNiF_3_, and TpTam‐KNiF_3_ samples exhibit mesopores with diameters of 10–30 nm (Figure 1A‐c,e,g; Figure S2, Supporting Information (the transmission electron microscopy (TEM) images)), whereas TpPa‐KCoF_3_, TpBpy‐KCoF_3_, and TpTam‐KCoF_3_ feature macropores ranging from 50 to 300 nm (Figure 1A‐d,f,h). In both cases, the pore sizes in the polymers closely correspond to the particle sizes of the perovskite fluorides; the uses of KNiF_3_ and KCoF_3_ lead to the formation of pores with a size of 10–30 nm and 50–300 nm, respectively. The pore size distributions (Figure S3, Supporting Information) and average pore sizes (**Table** **1**) are determined from TEM images for the KNiF_3_‐templated samples (Figure S2, Supporting Information) and from STEM images for the KCoF_3_‐templated samples (Figure 1A). The samples synthesized using the same fluoride templates exhibit consistent pore size distributions and comparable average pore sizes. Additionally, TEM images of representative intermediate samples before the removal of K*M*F_3_ (Figure S4, Supporting Information) reveal that no porous structure has yet formed, and that polymeric moieties appear to coat the surfaces of the K*M*F_3_ particles. In the absence of a template, all reactions result in the formation of bulk particles (Figure S5a–c, Supporting Information). These findings indicate that the solid‐state Schiff reaction with perovskite fluorides effectively generates meso‐ and macro‐porous structures, confirming that perovskite fluorides function as water‐soluble templates. If polymerization occurs as a dense coating over the K*M*F_3_ surface, template removal would be inhibited. However, the complete removal of K*M*F_3_ results in the formation of well‐defined porous structures, suggesting that the polymer networks formed on the K*M*F_3_ surfaces are not so solid and contain sufficient voids to facilitate template removal. Unlike conventional templating strategies that involve liquid‐ or gas‐phase reactions, this approach uniquely employs a solvent‐free solid‐state reaction. Interestingly, TpDq‐K*M*F_3_ samples, which are obtained from the reaction of 2,4,6‐triformylphloroglucinol (Tp) with 2,6‐diaminoanthraquinone (Dq) in the presence of KNiF_3_ and KCoF_3_, do not exhibit continuous porous structures (Figure S6a,b, Supporting Information), although their morphologies differ from those of TpDq‐solid obtained without K*M*F_3_ nanoparticles (Figure S5d, Supporting Information). While the exact reason remains unclear, the high molecular weight and rigid structure of Dq may hinder polymerization within the voids between perovskite fluoride particles.

**Table 1:** BET specific surface areas, band gaps, and valence band maximum (VBM) positions of the samples.

Sample names	Average pore size^a)^ [nm]	BET specific surface area^b)^ [m^2^ g^−1]^	Band gap^c)^ [eV]	VBM positions^d)^ [eV]
TpPa‐KNiF_3_	22.55	297	2.21	5.55
TpPa‐KCoF_3_	70.09	151	2.19	5.56
TpPa‐solid	‐	109	2.17	5.52
TpBpy‐KNiF_3_	21.41	92	2.24	5.62
TpBpy‐KCoF_3_	86.84	178	2.24	5.59
TpBpy‐solid	‐	53	2.27	5.75
TpTam‐KNiF_3_	19.35	885	2.57	5.77
TpTam‐KCoF_3_	87.08	452	2.58	5.70
TpTam‐solid	‐	164	2.57	5.56

^a)^ calculated from the pore sizes in the TEM/STEM results;

^b)^ calculated from the N_2_ adsorption isotherms;

^c)^ calculated from the UV–vis spectra;

^d)^ calculated from the PYSA spectra, and based on the vacuum level.

Structural variations were observed among the TpPa‐KCoF_3_, TpBpy‐KCoF_3_, and TpTam‐KCoF_3_ samples (Figure 1A‐d,f,h). TpBpy‐KCoF_3_ exhibits relatively rough surfaces, whereas TpTam‐KCoF_3_ displays well‐defined porous structures. TpPa‐KCoF_3_ appears to be an intermediate case between the two. These differences can be attributed to variations in molecular rigidity and the number of reaction sites among the three amines. The reaction between Tp and Tam may proceed more rapidly due to the flexible structure of Tam and its four reactive sites, leading to the formation of dense walls. Conversely, the greater molecular rigidity and fewer reactive sites in Pa and Bpy may result in relatively rough pore surfaces in TpPa‐KCoF_3_ and TpBpy‐KCoF_3_. The particularly rough structure of TpBpy‐KCoF_3_ compared to TpPa‐KCoF_3_ may be explained by the slower reaction rate between Tp and Bpy, potentially due to intermolecular interactions within the Bpy molecular crystal, which are induced by its two phenyl groups. Because the relationship between the monomer structures and the resulting porous morphologies remains unclear, a more detailed mechanistic understanding will require further investigation.

The X‐ray diffraction (XRD) patterns of the obtained porous samples are shown in Figure S7 (Supporting Information). All samples synthesized with K*M*F_3_ exhibit amorphous structures. The XRD pattern of TpPa‐solid displays a broad peak at 4°–6° (Figure S8a, Supporting Information), indicating the formation of a crystalline covalent organic framework (COF), consistent with previous reports.^[^ ^36^ ^][^ In contrast, TpPa‐KNiF_3_ and TpPa‐KCoF_3_ exhibit fully amorphous structures. The confined spaces between fluoride nanoparticles in the K*M*F_3_‐templated reactions likely further restrict molecular mobility, preventing the bond formation–deformation process required for crystallization and resulting in amorphous polymer structures. For the TpBpy and TpTam series (Figure S8b,c, Supporting Information), all samples, regardless of the presence of K*M*F_3_, exhibit amorphous structures. While solvothermal synthesis^[^ ^38^ ^]^ and solid‐state reactions with solid acids^[^ ^39^ ^]^ have been reported to induce COF crystallization in TpBpy, simple mixing of the two monomers does not lead to crystallization. Moreover, no crystalline COF composed of Tp and Tam has been reported to date,^[^ ^40^ ^]^ making the amorphous structure of the TpTam series a reasonable outcome. Consequently, all meso‐ and macro‐porous organic polymers obtained in this study exhibit amorphous polymer walls.

The N_2_ adsorption/desorption isotherms and Barrett‐Joyner‐Halenda (BJH) pore size distributions of the porous samples are presented in Figure 1B,C, with the Brunauer–Emmett–Teller (BET) specific surface areas listed in Table 1. The isotherms of the porous samples obtained with KNiF_3_ (Figure 1B: TpPa‐KNiF_3_, TpBpy‐KNiF_3_, and TpTam‐KNiF_3_) exhibit hysteresis at relative pressures of 0.5–1.0, indicating the presence of cage‐type bottleneck mesopores.^[^ ^41^ ^]^ Among the three, only TpTam‐KNiF_3_ possesses a noticeable uptake at *P*/*P*_0_ ∼ 0.95, suggesting the presence of interparticle macropores. In the case of the samples obtained with KCoF_3_, TpPa‐KCoF_3_ and TpBpy‐KCoF_3_ show an adsorption uptake from a relative pressure of 0.8 due to macropore filling, accompanied by small hysteresis. On the other hand, TpTam‐KCoF_3_ exhibits large hysteresis with sharp desorption via cavitation at *P*/*P*_0_ ∼ 0.5, suggesting the presence of cage‐type bottleneck macropores with relatively small pathways between the macropores.^[^ ^41^ ^]^ This phenomenon likely arises from the denser polymer walls formed by Tp and Tam on the KCoF_3_ surfaces, consistent with the STEM observations discussed earlier, which may restrict the diffusion of N_2_ molecules through the pore network.

The samples synthesized without K*M*F_3_ (Tp*Xxx*‐solid) display low adsorption volumes and no distinct uptake at *P*/*P*_0_ > 0.5 (Figure S9, Supporting Information), indicating that K*M*F_3_ is crucial for generating meso‐ and macro‐porous structures in the organic polymers. Notably, although TpPa‐solid exhibits a crystalline structure, it still shows low N_2_ adsorption volume. This observation aligns with previous reports, which attribute the reduced adsorption to pore blockage by precursor reagents or oligomeric species.^[^ ^36^ ^]^

Interestingly, the isotherms of TpPa‐KNiF_3_, TpBpy‐KNiF_3_, TpPa‐KCoF_3_, and TpBpy‐KCoF_3_ show no uptake at low relative pressure (*P*/*P*₀ <0.1), whereas those of TpTam‐KNiF_3_ and TpTam‐KCoF_3_ exhibit clear micropore filling in this region (Figure 1B), indicating the presence of micropores despite their amorphous frameworks. Tam contains a greater number of reactive amine (─NH_2_) groups and has increased steric hindrance due to its bulky molecular structure compared to Pa and Bpy, which may promote micropore formation within the polymer walls. Indeed, TpTam‐solid, synthesized without a template, also exhibits micropore uptake (Figure S9c, Supporting Information), although to a lesser extent than TpTam‐KNiF_3_ and TpTam‐KCoF_3_. These results suggest that the solid‐state reactions of Tp and Tam in the presence of KNiF_3_ and KCoF_3_ lead to the formation of hierarchical porous materials with combined micro/mesopores and micro/macropores, respectively. A previous study reported that the solvothermal reaction between Tp and Tam resulted in amorphous but microporous compounds.^[^ ^40^ ^]^ The lower micropore content in TpTam‐solid may be attributed to restricted gas diffusion due to long, complex micropore networks in the bulk system or pore blockage by unreacted molecules.

The BET specific surface areas of the porous samples are higher than those of the corresponding bulk materials (Tp*Xxx*‐solid) (Table 1). In the TpPa and TpTam series, the samples synthesized with KNiF_3_ exhibit larger surface areas than those synthesized with KCoF_3_ due to the smaller pore size in the former. The TpTam series exhibits particularly high surface areas due to the presence of micropores. The BJH pore size distributions of porous polymer samples obtained with KNiF_3_ and KCoF_3_ (Figure 1C) confirm mesopore sizes of 10–30 nm and macropore sizes of 50–300 nm, respectively, which are consistent with the particle sizes of the K*M*F_3_ templates and the pore sizes observed in STEM images (Figure 1A).

For TpBpy samples, the BET specific surface area of TpBpy‐KCoF_3_ is larger than that of TpBpy‐KNiF_3_. The samples after N_2_ adsorption/desorption measurements reveal structural collapse in TpBpy‐KNiF_3_ due to pre‐heating at 100°C (Figure S10, Supporting Information), which may have contributed to the reduction in surface area. Similar to TpDq, the rigid structure of Bpy may limit polymerization, resulting in lower thermal stability for TpBpy‐KNiF_3_. While the colors of TpPa and TpTam series samples remain unchanged before and after pre‐heating for N_2_ adsorption/desorption measurements, TpBpy‐KNiF_3_ and TpBpy‐KCoF_3_ samples darkened (Figure S11, Supporting Information), suggesting further polymerization during pre‐heating, potentially leading to bandgap shrinkage. The higher structural retention of TpBpy‐KCoF_3_ compared to TpBpy‐KNiF_3_ is likely due to a slightly greater degree of polymerization during the solid‐state reaction. The larger particle size of KCoF_3_ may lead to larger interparticle voids, which can enhance the diffusion and collision frequency of reacted oligomeric species, thereby promoting more extensive polymerization.

To enhance polymerization in TpBpy‐KNiF_3_, heat treatment was performed before template removal. The N_2_ adsorption/desorption measurements and BET surface area analysis of the heat‐treated TpBpy‐KNiF_3_ sample (Figure S12, Supporting Information) reveal a high specific surface area with micropore uptake. However, this increase is attributed to the crystallization of TpBpy with micropores rather than improved structural retention due to higher polymerization. Indeed, STEM images of heat‐treated TpBpy‐KNiF_3_ (Figure S13, Supporting Information) show the presence of plate‐ or needle‐like crystals. These results suggest that achieving a higher degree of polymerization during the solid‐state reaction is essential for retaining mesoporous structures.

The N_2_ adsorption/desorption measurements and BET surface areas of TpDq‐KNiF_3_ and TpDq‐KCoF_3_ (Figure S14 and Table S1, Supporting Information) indicate that these samples are nearly non‐porous, consistent with STEM observations (Figure S6, Supporting Information). The N_2_ adsorption/desorption isotherms differ from those of TpDq‐solid (Figure S9d, Supporting Information), also suggesting that the presence of K*M*F_3_ influences particle size and morphology in the solid‐state reaction, even though meso‐ or macro‐pores do not form.

**Figure 2:**
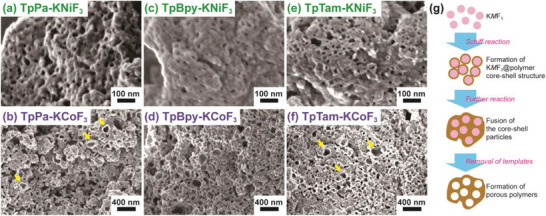
SEM images of a) TpPa‐KNiF_3_, b) TpPa‐KCoF_3_, c) TpBpy‐KNiF_3_, d) TpBpy‐KCoF_3_, e) TpTam‐KNiF_3_, f) TpTam‐KCoF_3_, and g) a proposed pathway for the formation of organic porous polymers with K*M*F_3_. The yellow arrows in (b, f) show hollow particles, which may be obtained from core–shell intermediates.

The surface characteristics of the porous samples are crucial, as the accessibility of reagents to the pores depends on the degree of pore openness. To investigate this, SEM observations were conducted (**Figure** **2**). In all porous samples, the particle surfaces exhibit open pores with sizes corresponding to those of the K*M*F_3_ particles used as templates. However, the density of these open pores is lower than that of porous materials synthesized using polymer micelles as templates,^[^ ^42^, ^43^, ^44^ ^]^ and some closed regions appear to be composed of aggregated polymer particles. Based on these observations, we propose the following formation mechanism (Figure 2g): i) K*M*F_3_ surfaces are coated with polymerized moieties, forming core–shell‐like structures, ii) these core–shell‐like particles fuse through additional polymerization, and iii) after template removal, meso‐ or macro‐pores are generated. Notably, hollow‐like particles with broken shells are observed, particularly in samples synthesized with KCoF_3_, as indicated by yellow arrows in Figure 2b,f. These observations support the proposed first step of the mechanism, suggesting a possible interaction between the K*M*F_3_ surfaces and the precursor molecules. While the exact nature of this interaction remains unclear, certain amines and aldehydes may engage in weak coordination with exposed metal cations on the K*M*F_3_ surface, facilitating the initial stages of polymerization at the template interface. The fusion of core–shell particles leads to the formation of bottle‐necked porous structures, contributing to the relatively large hysteresis observed in the N_2_ adsorption/desorption measurements.

**Figure 3:**
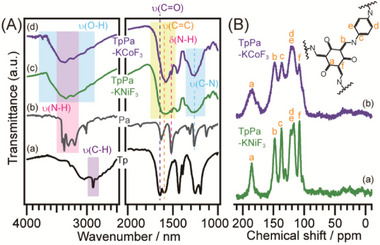
A) IR spectra of (a) Tp, (b) Pa, (c) TpPa‐KNiF_3_, and (d) TpPa‐KCoF_3_, and B) ^13^C CP/MAS NMR spectra of (a) TpPa‐KNiF_3_ and (b) TpPa‐KCoF_3_.

The FT–IR spectra of TpPa‐KNiF_3_ and TpPa‐KCoF_3_ (**Figure** **3**A) exhibit broad bands in the ranges of 1800–1500 and 1400–1100 cm^−1^, corresponding to merged υ(C═O) and υ(C═C) vibrations and broad υ(C─N) vibrations, respectively. Additionally, a significant decrease in the υ(N─H) and δ(N─H) vibration peaks of Pa, along with the absence of the υ(C─H) vibration peak from Tp, indicates the successful Schiff reaction between the aldehyde groups of Tp and the amino groups of Pa. The spectra closely resemble those of the keto‐form of TpPa,^[^ ^45^ ^]^ suggesting that keto‐enol tautomerization occurs even in the solid‐state reaction, consistent with previous reports.^[^ ^36^ ^]^ The FT–IR spectra of the solid‐state reaction product without K*M*F_3_ (TpPa‐solid) and the solvothermally synthesized product (TpPa‐solvo) (Figure S15, Supporting Information) are nearly identical to those of the porous samples (TpPa‐KNiF_3_ and TpPa‐KCoF_3_), indicating that the chemical bonding in all samples is similar. The only notable difference is in the band intensities around 3500 cm^−1^, attributed to water content. The porous samples exhibit stronger absorption in this region, suggesting a higher water content, likely due to the presence of unreacted functional groups on their hydrophilic, amorphous surfaces. Furthermore, solid‐state ^13^C MAS NMR spectra of TpPa‐KNiF_3_ and TpPa‐KCoF_3_ (Figure 3B) show very broad peaks, confirming polymerization via the Schiff reaction. In contrast, the crystalline COF sample obtained by solvothermal reaction exhibits sharp peaks in its ^13^C MAS NMR spectrum (Figure S16, Supporting Information), indicating that the broadness of the peaks in the K*M*F_3_‐templated samples results from their amorphous frameworks. The relative compositional ratios of the TpPa‐series samples, as determined by X‐ray Photoelectron Spectroscopy (XPS), are summarized in Table S2 (Supporting Information). The samples synthesized via solid‐state reaction, both with and without K*M*F_3_, exhibit lower nitrogen content and higher oxygen content compared to the theoretical values expected for an ideal crystalline COF structure. In contrast, TpPa‐solvo, which possesses a crystalline framework, shows elemental ratios closely matching the ideal composition. These results suggest that the incorporation of Pa molecules is less efficient under solid‐state reaction conditions at room temperature, leading to a lower degree of polymerization. The polymerization via the Schiff reaction in the TpBpy and TpTam series is also confirmed by IR spectra (Figures S17 and S18, Supporting Information).

**Figure 4:**
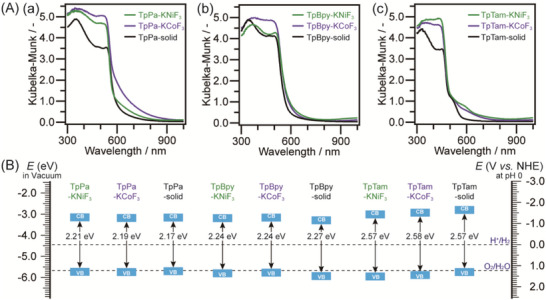
A) UV–vis DR spectra of (a) TpPa, (b) TpBpy, and (c) TpTam series samples. B) Band structures of the obtained samples on the basis of the band gap and the position of valence band maximum (VBM) derived from UV–vis DR and PYSA spectra, respectively. CB and VB mean conduction and valence bands, respectively.

The obtained samples are semiconductive compounds; therefore, we investigated their properties. **Figure** **4**A presents the UV–vis diffuse reflectance (UV–vis DR) spectra of the porous samples along with those of the samples synthesized without templates. All samples exhibit visible‐light absorption. The band gaps are estimated from the UV–vis DR spectra using Tauc plots (Figures S19–S21, Supporting Information; Table 1). Additionally, photoemission yield spectroscopy in air (PYSA) is performed to determine the valence band maximum (VBM) positions relative to the vacuum level (Figures S22–S24, Supporting Information; Table 1). Combining the band gap values from UV–vis DR spectra with the VBM positions from PYSA spectra, the band structures of the samples are constructed (Figure 4B). The results indicate that the band gaps of the porous samples are nearly identical to those of the corresponding non‐porous samples, suggesting that the presence of fluorides in the solid‐state reaction does not significantly alter the polymerization process. Comparing the band structures with relevant redox potentials, we find that these materials possess energy levels suitable for photocatalytic water reduction (hydrogen evolution reaction), CO_2_ reduction, and hydrogen peroxide production. According to previous reports involving DFT calculations on Tp*Xxx* polymers,^[^ ^46^ ^]^ charge separation in Tp*Xxx*‐based systems is facilitated by an excited‐state intramolecular proton transfer (ESIPT) mechanism. In this process, a portion of the keto groups in the polymer backbone is converted to enol forms upon light irradiation, where the enol–imine and keto–enamine segments function as electron donor and acceptor, respectively, to promote charge separation. Owing to this mechanism, these compounds are well‐suited for use as photocatalysts.

To evaluate the effectiveness of porous organic polymers, we investigated their sacrificial photocatalytic hydrogen evolution under visible‐light irradiation. Among the various sample series, the TpPa series (TpPa‐KNiF_3_, TpPa‐KCoF_3_, and TpPa‐solid) is selected for evaluation due to its combination of broad visible‐light absorption and well‐defined porous structures. Additionally, TpPa_solvo, a solvothermally synthesized crystalline microporous material derived from the same monomers, is included for comparison. TpPa_solvo exhibits a very high specific surface area (524 m^2^ g^−1^, Figure S25, Supporting Information) and a band structure similar to the other TpPa samples (Figures S22d and S26, Supporting Information). Platinum co‐catalysts (3.0 wt.%) were deposited onto all four samples via photodeposition. For photocatalytic evaluation, 5 mg of TpPa‐KNiF_3_ and TpPa‐KCoF_3_ and 10 mg of TpPa‐solid and TpPa‐solvo were used, as TpPa‐solid and TpPa‐solvo exhibit lower dispersibility in water, necessitating a larger powder quantity. The hydrogen evolution reactions were conducted in a solution containing 50 mm phosphate buffer and 2 mm sodium ascorbate under visible‐light irradiation with λ >420 nm (the typical gas chromatography charts are shown in Figure S27, Supporting Information).

**Figure 5:**
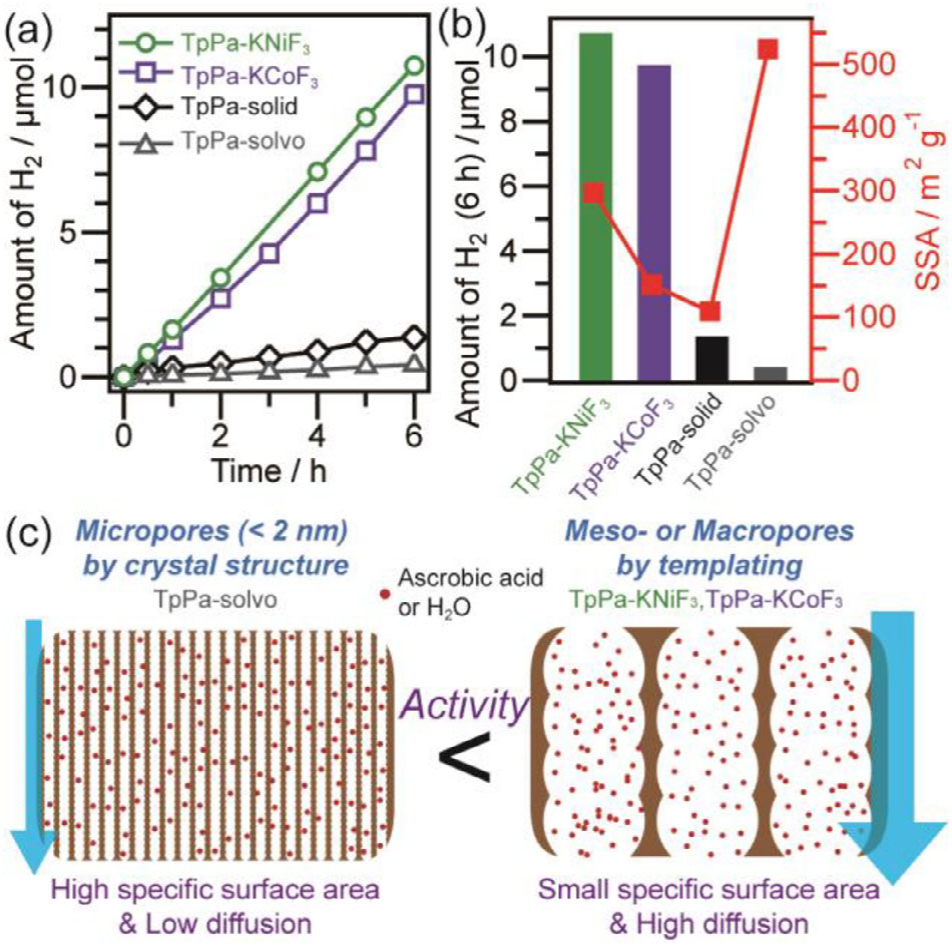
a) Time courses of photocatalytic hydrogen evolution under visible‐light irradiation for the TpPa series samples, b) correlation between the amount of hydrogen evolved for 6 h and the specific surface areas of the samples, and c) schematic illustration of the relationship between surface area and molecular diffusion.

The hydrogen evolution rates of TpPa‐KNiF_3_ and TpPa‐KCoF_3_ are significantly higher than those of TpPa‐solid and TpPa‐solvo (**Figure** **5**a). The low activity of TpPa‐solid is attributed to its low specific surface area (Figure 5b). Although TpPa‐solvo has an extremely high surface area (Figure S25, Supporting Information), its photocatalytic activity was much lower than that of TpPa‐KNiF_3_ and TpPa‐KCoF_3_ (Figure 5b). The high surface area of TpPa‐solvo originates from its microporous crystalline structure (Figure S28, Supporting Information), with micropore sizes of ≈1.8 nm.^[^ ^36^, ^45^ ^]^ However, these small pores likely hinder reagent diffusion, leading to reduced catalytic activity. In contrast, the meso‐ and macro‐porous structures of TpPa‐KNiF_3_ and TpPa‐KCoF_3_ facilitate efficient reagent diffusion, resulting in higher catalytic performance (Figure 5c). These findings indicate that micropores in COF materials are generally ineffective for catalytic applications due to restricted molecular diffusion. Conversely, meso‐ and macro‐porous organic polymers significantly enhance catalytic efficiency, demonstrating their potential for photocatalytic applications. The photocatalytic activity of TpPa‐KNiF_3_ is higher than that of TpPa‐KCoF_3_, which can be attributed to its larger specific surface area. However, the difference in activity is relatively small compared to the difference in their surface areas. This suggests that the enhanced molecular diffusion enabled by the macropores (50–300 nm) in TpPa‐KCoF_3_ significantly contributes to catalytic performance, compensating for its lower surface area relative to the mesoporous TpPa‐KNiF_3_. These findings highlight that achieving an optimal balance between surface area and pore size is crucial for enhancing catalytic activity.

XPS measurements were conducted on TpPa‐KNiF_3_ and TpPa‐KCoF_3_ before and after photocatalytic evaluation to investigate the effects of the catalytic reaction on the chemical states of the catalysts (Figure S29, Supporting Information). The C 1s spectrum of TpPa‐KCoF_3_ after photocatalysis (Figure S29B‐a, Supporting Information) shows a new peak at 284.8 eV, likely corresponding to C─C bonding from ascorbic acid or its decomposition products. Although this peak is not clearly observed in the spectrum of TpPa‐KNiF_3_ (Figure S29A‐a, Supporting Information), the broad nature of the signal suggests that similar contributions from ascorbic acid or its derivatives may also be present. Therefore, the C 1s spectra do not allow for a precise evaluation of the catalyst's chemical state. In contrast, nitrogen species are not introduced during the photocatalytic process. As shown in the N 1s spectra of TpPa‐KNiF_3_ and TpPa‐KCoF_3_ before and after the reaction (Figure S29A‐b,B‐b, Supporting Information), no significant changes are observed, indicating that the nitrogen‐containing polymer framework remains chemically stable under the reaction conditions. Furthermore, the steady increase in hydrogen evolution during photocatalytic testing supports the structural and catalytic stability of the polymer‐based materials.

All meso‐ and macro‐porous compounds presented in this study possess disordered porous networks, in which some pore pathways may be partially blocked or inaccessible, potentially limiting effective diffusion. In contrast, highly ordered porous structures could promote more efficient mass transport, offering a promising strategy for further enhancing catalytic activity. The development of highly ordered porous structures using water‐soluble templates will be explored in future work.

## 3 Conclusion

This study demonstrates the use of perovskite fluorides (K*M*F_3_) as water‐soluble templates for the synthesis of meso‐ and macro‐porous organic polymers. Solid‐state polymerization via the Schiff reaction between aldehydes and amines in the presence of K*M*F_3_, followed by simple water treatment to remove the template, successfully yields porous polymers. By tuning the size of K*M*F_3_ particles, the pore size of the resulting polymers is controlled from the mesoscale to the macroscale. Furthermore, this synthetic approach is applicable to various aldehyde–amine combinations, with the specific combination influencing the semiconductive properties of the resulting polymers. These findings indicate that this method enables the design of porous polymers with tailored pore sizes and semiconductive characteristics. To highlight the advantages of meso‐ and macro‐porous polymers, photocatalytic hydrogen evolution reactions are performed using the synthesized porous materials. The catalytic activity is significantly higher than that of a crystalline microporous COF with a much higher surface area but composed of the same molecular components. This enhanced activity is attributed to the improved diffusion of reactants and products facilitated by larger pores, strongly supporting the idea that meso‐ and macro‐porosity enhances catalytic performance. The use of K*M*F_3_ as a water‐soluble template offers a versatile platform for synthesizing meso‐ and macro‐porous materials with diverse compositions, many of which are challenging to achieve using conventional methods. This approach has the potential to expand the scope of porous materials chemistry, opening new possibilities for applications in catalysis, separation, and optoelectronics.

## 4 Experimental Section

### Solid State Polymerization of Aldehydes and Amines

First, aldehyde‐containing molecules were mixed with a large amount of K*M*F_3_ particles, with a small amount of ethanol added to facilitate homogeneous mixing. Once uniform, the mixtures were further combined with amine‐containing molecules and stirred for 45 min. The resulting products were washed with acetone and dichloromethane to remove unreacted molecules. Finally, the washed samples were treated with water to dissolve the K*M*F_3_ particles, yielding meso‐ or macro‐porous organic polymers. 2,4,6‐Triformylphloroglucinol (Tp) was used as the aldehyde precursor, while p‐phenylenediamine (Pa), [2,2'‐bipyridine]‐5,5'‐diamine (Bpy), 2,6‐diaminoanthraquinone (Dq), and tetrakis(4‐aminophenyl)methane (Tam) were selected as amine counterparts. For comparison, Tp was reacted with amine molecules in the solid state without K*M*F_3_ under identical conditions. The resulting samples were denoted as Tp*Xxx*‐solid.

### Photocatalytic Hydrogen Evolution Over TpPa‐KNiF_3_, TpPa‐KNiF_3_, TpPa‐solid, and TpPa‐solvo

Sacrificial H_2_ evolution was conducted using a top‐window reaction vessel connected to a gas‐closed circulation system. Pt as a cocatalyst for H_2_ evolution was deposited by an in situ photoreduction method using H_2_PtCl_6_·6H_2_O as a precursor. Appropriate amounts of samples (TpPa‐KNiF_3_, TpPa‐KCoF_3_; 5 mg, TpPa‐solid and TpPa‐solvo; 10 mg) were dispersed in 160 mL of a 2 mM sodium L‐ascorbate and 50 mM phosphate buffer mixture solution (pH = 6.8) containing the cocatalyst source. The temperature of the reactant solution was kept at 293 K by circulation of cooling water. 10 kPa of Ar was introduced into the system after deaeration. The suspension was irradiated with visible light (λ >420 nm) using a 300 W Xe‐arc lamp (Excelitas, Cermax PE300BF) with a long pass filter. Evolved gas was analyzed using an online gas chromatograph (Shimadzu, GC‐8A with MS‐5A column, TCD detector, and Ar carrier).

## Conflict of Interest

The authors declare no conflict of interest.

## Supporting information



Supporting Information

## Data Availability

The data that support the findings of this study are available from the corresponding author upon reasonable request.
