# Supplementary material for: A Universal Approach Using Water‐Soluble Templates for Meso‐ and Macro‐Porous Organic Polymers
Source: Adv Sci (Weinh). 2025 Jul 22;12(39):e08489. doi: 10.1002/advs.202508489 (PMC12533404; doi:10.1002/advs.202508489)
Supplement: Supplementary file 1 — Supporting Information [file ADVS-12-e08489-s001.pdf]

ADVANCED SCIENCE
Open Access

Supporting Information

for *Adv. Sci.*, DOI 10.1002/advs.202508489

A Universal Approach Using Water-Soluble Templates for Meso- and Macro-Porous Organic Polymers

Yusuke Asakura, Steven Adiwijaya, Shunya Yoshino, Hideki Kato and Yusuke Yamauchi**

Supporting Information

A Universal Approach Using Water-Soluble Templates for Meso- and Macro-porous Organic Polymers

Yusuke Asakura[a,*], Steven Adiwijaya[a], Shunya Yoshino[b], Hideki Kato[b], Yusuke Yamauchi[a,c,*]

[a]Department of Materials Process Engineering, Graduate School of Engineering, Nagoya University, Furo-cho, Chikusa-ku, Nagoya, Aichi 464-8603, Japan

[b]Institute of Multidisciplinary Research for Advanced Materials, Tohoku University, 2-1-1, Katahira, Aoba-ku, Sendai, Miyagi 980-8577, Japan

[c]Australian Institute for Bioengineering and Nanotechnology (AIBN), The University of Queensland, Brisbane, Queensland 4072, Australia

Email: asa.y@nagoya-u.jp, y.yamauchi@uq.edu.au

Contents

Experimental 4

Figure S1. XRD patterns of (a) $KNiF_3$ and (b) $KCoF_3$ with standard patterns ($KNiF_3$: JCPDS No. 01-076-2392, $KCoF_3$: JCPDS No. 00-018-1006) 7

Figure S2. TEM images of (a) TpPa-$KNiF_3$, (b) TpBpy-$KNiF_3$, and (c) TpTam-$KNiF_3$ 8

Figure S3. Pore size distribution of (a) TpPa-$KNiF_3$, (d) TpPa-$KCoF_3$, (e) TpBpy-$KNiF_3$, (f) TpBpy-$KCoF_3$, (g) TpTam-$KNiF_3$, and (h) TpTam-$KCoF_3$. 9

Figure S4. TEM images of the intermediates before the removal of KMF_3 for (a) TpPa-$KNiF_3$ and (b) TpPa-$KCoF_3$ 10

Figure S5. STEM images of (a) TpPa-solid, (b) TpBpy-solid, (c) TpTam-solid, and (d) TpDq-solid. 11

Figure S6. STEM images of TpDq-$KNiF_3$ and TpDq-$KCoF_3$. 12

Figure S7. XRD patterns of (a) TpPa-$KNiF_3$, (b) TpPa-$KCoF_3$, (c) TpBpy-$KNiF_3$, (d) TpBpy-$KCoF_3$, (e) TpTam-$KNiF_3$, and (f) TpTam-$KCoF_3$. 13

Figure S8. XRD patterns of (a) TpPa-solid, (b) TpBpy-solid, and (c) TpTam-solid. 14

Figure S9. N_2 adsorption/desorption isotherms of (a) TpPa-solid, (b) TpBpy-solid, (c) TpTam-solid, and (d) TpDq-solid. 15

Figure S10. A degraded part in the STEM image of TpBpy-$KNiF_3$ after the N_2 adsorption/desorption measurement. 16

Figure S11. Appearance of the porous polymers before and after the N_2 adsorption/desorption measurements. 17

Figure S12. (a) N_2 adsorption/desorption measurement and (b) BJH pore size distribution of TpBpy-$KNiF_3$ heated at 80 ºC before the removal of $KNiF_3$. 18

Figure S13. STEM image of Heated-TpBpy-$KNiF_3$. 19

Figure S14. N_2 adsorption/desorption measurement of (a) TpDq-$KNiF_3$ and (b) TpDq-$KCoF_3$. 20

Figure S15. IR spectra of (a) Tp, (b) Pa, (c) TpPa-$KNiF_3$, (d) TpPa-$KCoF_3$, (e) TpPa-solid, and (f) TpPa-solvo. 21

Figure S16. ^{13}C CP/MAS NMR spectrum of TpPa-solvo....22

Figure S17. IR spectra of (a) Tp, (b) Bpy, (c) TpBpy-$KNiF_3$, (d) TpBpy-$KCoF_3$, and (e) TpBpy-solid. 23

Figure S18. IR spectra of (a) Tp, (b) Tam, (c) TpTam-$KNiF_3$, (d) TpTam-$KCoF_3$, and (e) TpTam-solid. 24

Figure S19. Tauc plots of TpPa-$KNiF_3$, TpPa-$KCoF_3$, and TpPa-solid. 25

Figure S20. Tauc plots of TpBpy-$KNiF_3$, TpBpy-$KCoF_3$, and TpBpy-solid. 26

Figure S21. Tauc plots of TpTam-$KNiF_3$, TpTam-$KCoF_3$, and TpTam-solid. 27

Figure S22. PYSA spectra of (a) TpPa-$KNiF_3$, (b) TpPa-$KCoF_3$, (c) TpPa-solid, and (d) TpPa-solvo. 28

Figure S23. PYSA spectra of (a) TpBpy-$KNiF_3$, (b) TpBpy-$KCoF_3$, and (c) TpBpy-solid....29

Figure S24. PYSA spectra of (a) TpTam-$KNiF_3$, (b) TpTam-$KCoF_3$, and (c) TpTam-solid. 30

Figure S25. N_2 adsorption/desorption measurement of TpPa-solvo. 31

Figure S26. (a) UV-vis spectrum, (b) Tauc plot, and (c) Proposed band structure of TpPa-solvo. 32

Figure S27. Gas chromatography profile of photocatalytic hydrogen evolution evaluation for TpPa-$KCoF_3$ (a) under dark condition and under light irradiation for (b) 2 h and (c) 6 h. 33

Figure S28. XRD pattern of TpPa-solvo....34

Figure S29. XPS (a) C 1s and (b) N 1s spectra of (A) TpPa-$KNiF_3$ and (B) TpPa-$KCoF_3$ before and after photocatalytic hydrogen evolution reactions. 35

Table S1. BET surface areas of the TpDq series calculated from N_2 adsorption isotherms. 36

Table S2. Elemental compositions of the TpPa-series samples based on the XPS results....37

Experimental

Materials. Nickel (II) chloride ($NiCl_2 \cdot 6H_2O$), cobalt (II) chloride hexahydrate (98%, $CoCl_2 \cdot 6H_2O$, Sigma-Aldrich), Potassium fluoride dihydrate (98%, $KF \cdot 2H_2O$, Sigma-Aldrich), ethylene glycol (99.5%, Fujifilm Wako Pure Chemical Co.), and methanol (99.5%, Fujifilm Wako Pure Chemical Co.) were used for synthesis of perovskite fluoride without any purification. 2,4,6-Triformylphloroglucinol (> 98%, Tokyo Chemical Industry Co., Ltd.), 1,4-phenelyenediamine (> 98%, Tokyo Chemical Industry Co., Ltd.), [2,2'-Bipyridine]-5,5'diamine (> 98%, Tokyo Chemical Industry Co., Ltd.), Tetrakis(4-aminophenyl)methane (> 95%, Tokyo Chemical Industry Co., Ltd.), 2,6-Diaminoanthraquinone (> 97%, Tokyo Chemical Industry Co., Ltd.), acetone (99%, Fujifilm Wako Pure Chemical Co.), and dichloromethane (99%, Fujifilm Wako Pure Chemical Co.) were used for Schiff reactions and their post-treatments without any purification. Hydrogen hexachloroplatinate(IV) hexahydrate (99.0%, $H_2PtCl_6 \cdot 6H_2O$, Kanto Chemical Co., Inc.) and L-ascorbate (98.0%, Kanto Chemical Co., Inc.) were used for evaluating photocatalytic hydrogen evolution without any purification.

Solvothermal synthesis of perovskite fluorides. According to a previous report (H. Fan *et al.*, *J. Colloid Interface Sci.*, **557**, 546), K*M*F_3 particles were synthesized *via* a solvothermal method. $NiCl_2 \cdot 6H_2O$ or $CoCl_2 \cdot 6H_2O$ (6 mmol) and $KF \cdot 2H_2O$ (15 mmol) were dissolved in ethylene glycol (40 mL) under ultrasonic treatment. The resulting mixtures were then subjected to solvothermal treatment at 120 °C for $KNiF_3$ and 180 °C for $KCoF_3$. The reaction durations were 10 h for $KNiF_3$ and 20 h for $KCoF_3$ synthesis. After the reaction, the precipitates were collected, washed twice with methanol, and dried under reduced pressure.

Solid state polymerization of aldehydes and amines. First, aldehyde-containing molecules were mixed with a large amount of K*M*F_3 particles, with a small amount of ethanol added to facilitate homogeneous mixing. Once uniform, the mixtures were further combined with amine-containing molecules and

stirred for 45 min. The resulting products were washed with acetone and dichloromethane to remove unreacted molecules. Finally, the washed samples were treated with water to dissolve the K*M*F_3 particles, yielding meso- or macro-porous organic polymers. 2,4,6-Triformylphloroglucinol (Tp) was used as the aldehyde precursor, while p-phenylenediamine (Pa), [2,2'-bipyridine]-5,5'-diamine (Bpy), 2,6-diaminoanthraquinone (Dq), and tetrakis(4-aminophenyl)methane (Tam) were selected as amine counterparts. The obtained samples were designated as Tp*Xxx*-K*M*F_3, where *Xxx* represents the amine used (Pa, Bpy, Dq, or Tam) and *M* denotes the metal species in the perovskite fluoride (Ni or Co). For comparison, Tp was reacted with amine molecules in the solid state without K*M*F_3 under identical conditions. The resulting samples were denoted as Tp*Xxx*-solid.

Characterizations. Scanning transmission electron microscope (STEM) and scanning electron microscope (SEM) images were recorded on a Zeiss GeminiSEM 560 microscopy. STEM measurements were conducted for the samples on a TEM grid with a carbon layer, and SEM measurements were carried out after the samples on a silicon substrate were covered with an Os metal layer deposited by a Meiwafosis Neoc-STB coater. N_2 adsorption-desorption measurements were performed using a MicrotracBEL Bell Mini instrument. Powder X-ray diffraction (XRD) patterns were collected by a Rigaku SmartLab diffractometer with X-ray CuKa (l = 0.15148 nm) using para-focusing method. Fourier-transform infrared (FTIR) spectra were recorded using a JASCO FT/IR-4X spectrometer. Solid-state ^{13}C MAS NMR spectra were collected on a Bruker AVANCE NEO 500 spectrometer with a spinning frequency of 14 kHz. UV-vis spectra were collected with a JASCO V770 spectrometer. Valence band maximums (VBMs) of the samples are characterized on the basis of photoemission yield spectroscopy in air (PYSA) spectra recorded on a RIKEN KEIKI AC-2 spectrometer.

Solvothermal reaction between Tp and Pa. According to the previous reports (Kandambeth, S. *et al.*, *J. Am. Chem. Soc.* **2012**, 134 (48), 19524-19527), TpPa-solvo was synthesized. Tp and Pa were

dissolved in dimethylformamide and subjected to solvothermal treatment at 120 °C for 72 h. After the reaction, the resulting precipitate was washed with acetone, dichloromethane, and ethanol, and then dried under reduced pressure. The obtained sample was designated as TpPa-solvo.

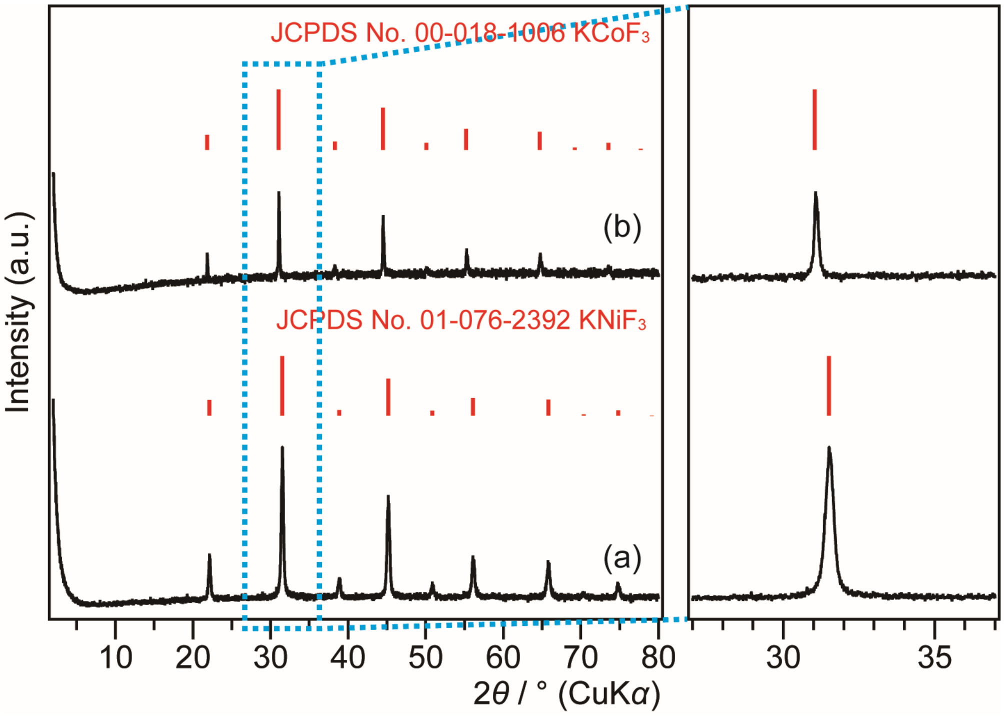

Figure S1. XRD patterns of (a) $KNiF_3$ and (b) $KCoF_3$ with standard patterns ($KNiF_3$: JCPDS No. 01-076-2392, $KCoF_3$: JCPDS No. 00-018-1006).

Comment for Figure S1: The XRD patterns of $KNiF_3$ and $KCoF_3$ are in good agreement with their respective standard reference patterns, confirming the successful formation of phase-pure materials.

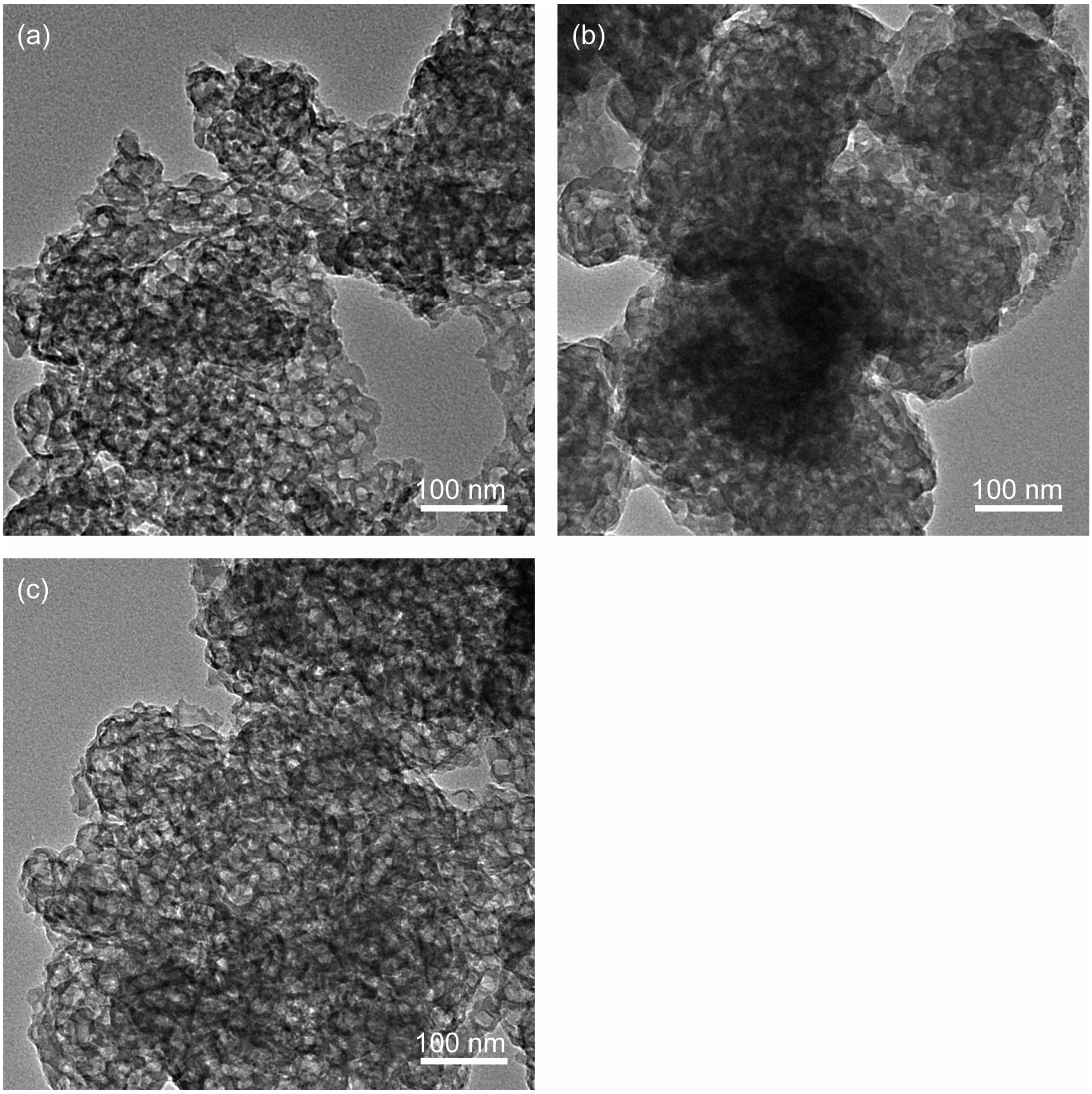

Figure S2. TEM images of (a) TpPa-$KNiF_3$, (b) TpBpy-$KNiF_3$, and (c) TpTam-$KNiF_3$.

Comment for Figure S2: The TEM images show clear mesoporous structures in TpPa-$KNiF_3$, TpBpy-$KNiF_3$, and TpTam-$KNiF_3$.

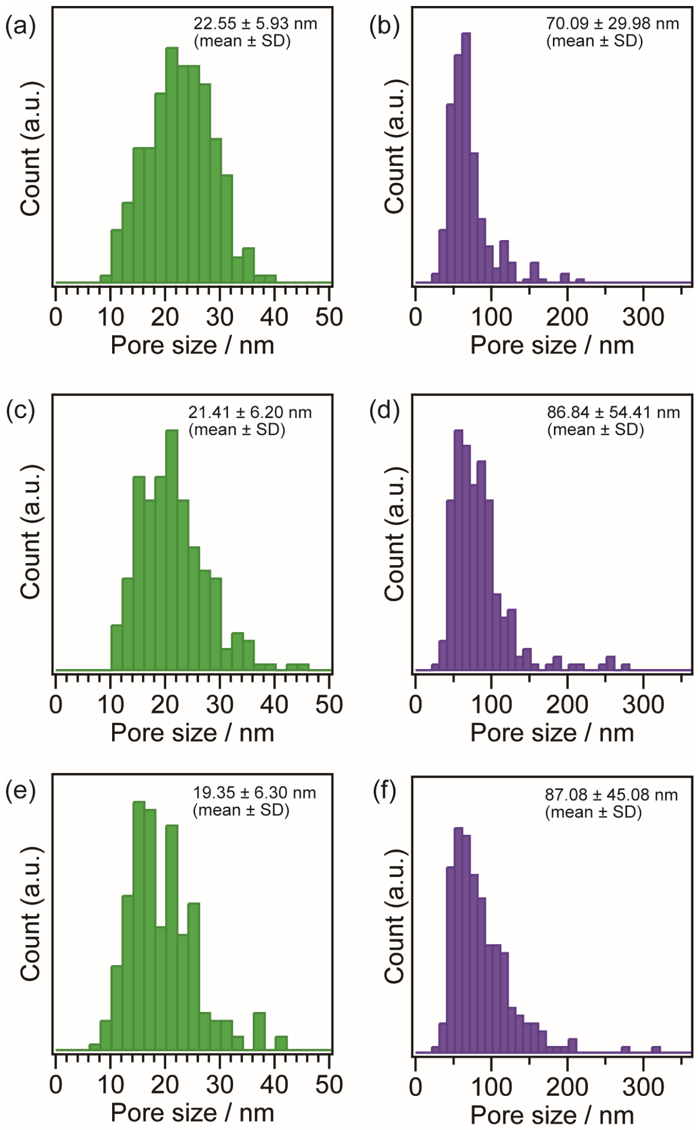

Figure S3. Pore size distribution of (a) TpPa-$KNiF_3$, (d) TpPa-$KCoF_3$, (e) TpBpy-$KNiF_3$, (f) TpBpy-$KCoF_3$, (g) TpTam-$KNiF_3$, and (h) TpTam-$KCoF_3$. They are collected from the TEM images for the case of $KNiF_3$ and from the STEM images for the case of $KCoF_3$.

Comment for Figure S3: The samples synthesized using the same fluoride templates exhibit similar pore size distributions.

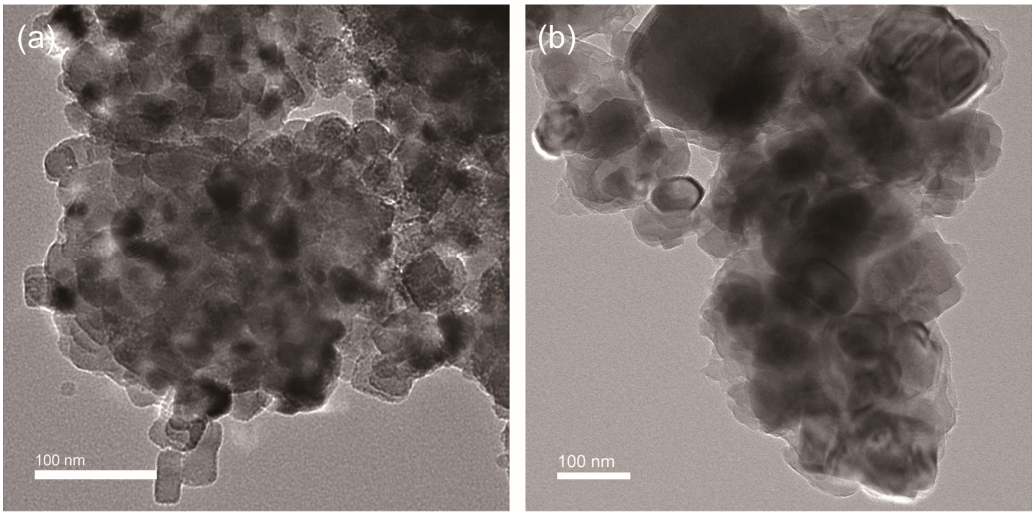

Figure S4. TEM images of the intermediates before the removal of KMF_3 for (a) TpPa-$KNiF_3$ and (b) TpPa-$KCoF_3$.

Comment for Figure S4: The TEM images of the intermediate samples before the removal of $K\mathit{M}F_3$ reveal that no porous structure has yet formed, and that polymeric moieties appear to coat the surfaces of the $K\mathit{M}F_3$ particles.

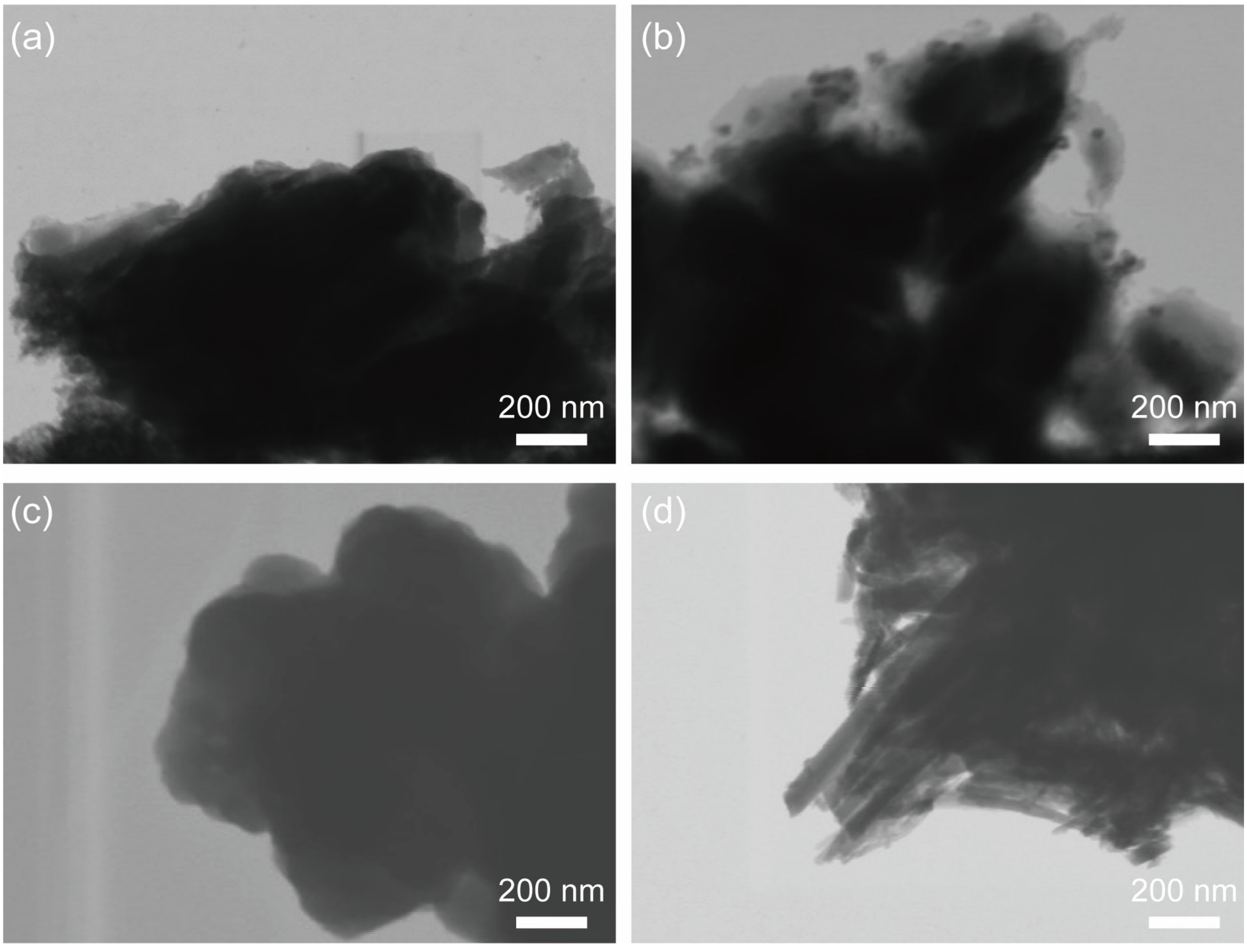

Figure S5. STEM images of (a) TpPa-solid, (b) TpBpy-solid, (c) TpTam-solid, and (d) TpDq-solid.

Comment for Figure S5: The SEM images of the samples synthesized without KMF_3 reveal non-porous morphologies across all molecular combinations examined (TpPa, TpBpy, TpTam, and TpDq).

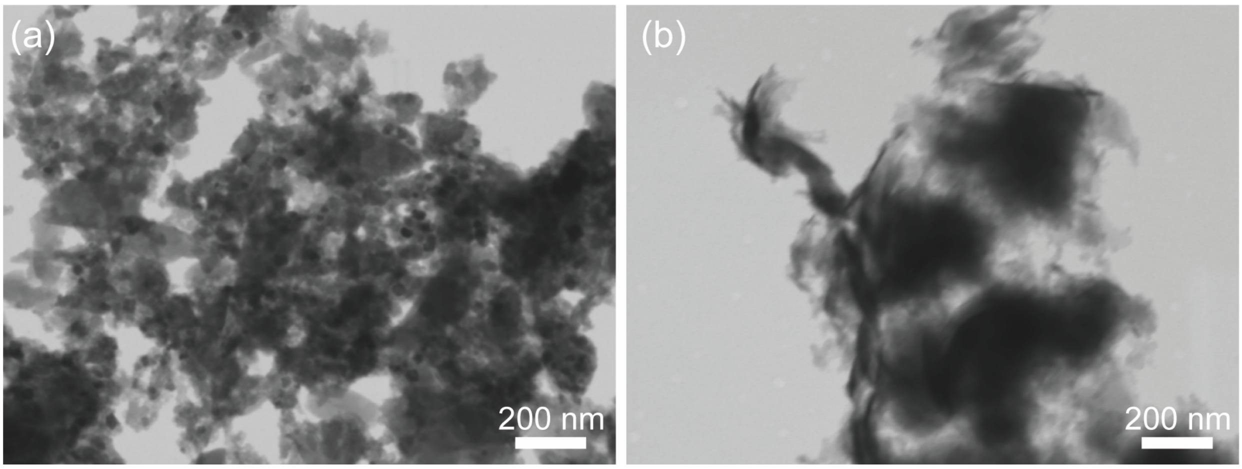

Figure S6. STEM images of TpDq-$KNiF_3$ and TpDq-$KCoF_3$.

Comment for Figure S6: SEM images of TpDq-$KNiF_3$ and TpDq-$KCoF_3$ exhibit non-porous morphologies, indicating that KMF_3 does not function effectively as a template for the formation of porous TpDq structures.

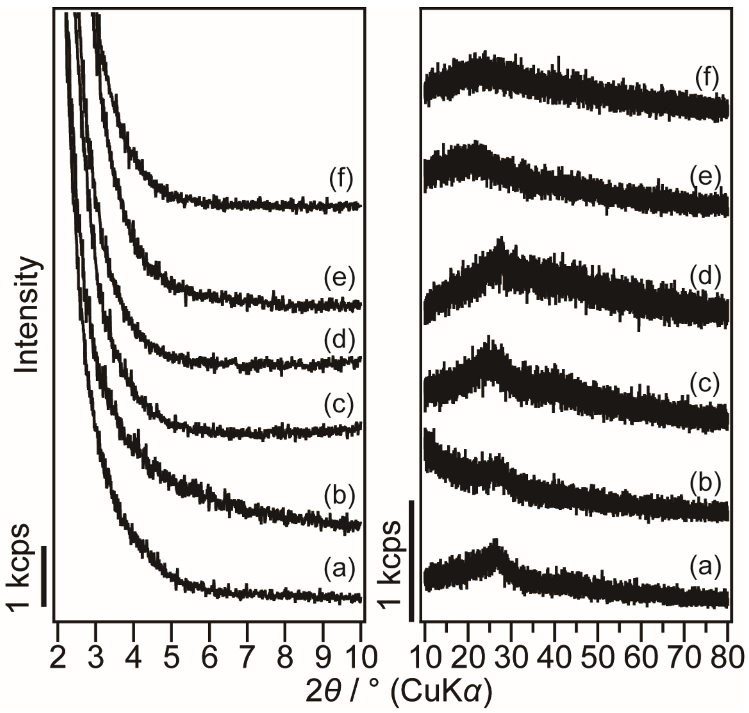

Figure S7. XRD patterns of (a) TpPa-$KNiF_3$, (b) TpPa-$KCoF_3$, (c) TpBpy-$KNiF_3$, (d) TpBpy-$KCoF_3$, (e) TpTam-$KNiF_3$, and (f) TpTam-$KCoF_3$. The left and right images show low- and high-angle regions, respectively.

Comment for Figure S7: The XRD patterns indicate that all the porous polymers possess amorphous frameworks.

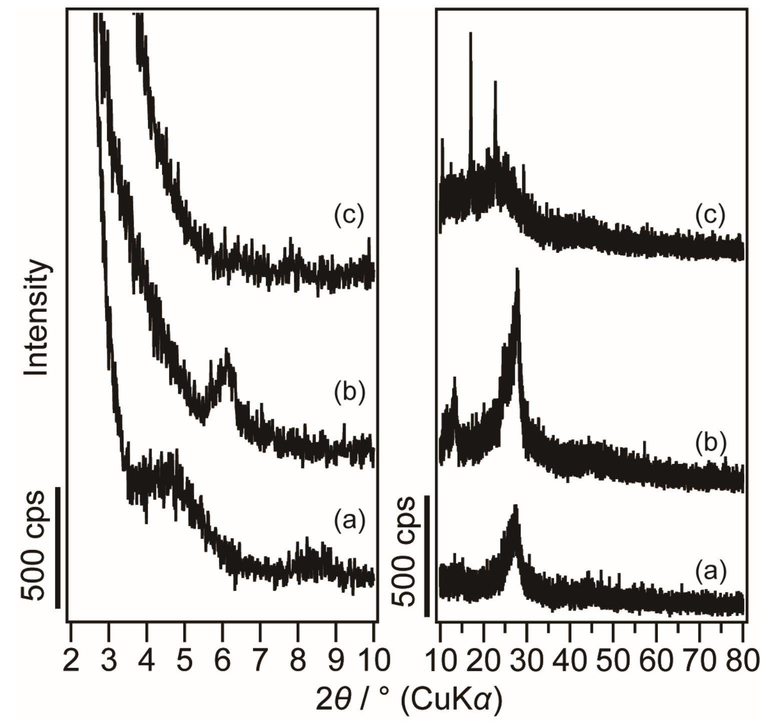

Figure S8. XRD patterns of (a) TpPa-solid, (b) TpBpy-solid, and (c) TpTam-solid. The left and right images show low- and high-angle regions, respectively.

Comment for Figure S8: The pattern of TpPa-solid shows a broad peak at 4-6°, meaning the formation of crystalline COF. This behavior is similar to the previous reports (Biswal, B. P. *et al.*, *J. Am. Chem. Soc.* **2013**, *135* (14), 5328-5331.). Although TpBpy-solid exhibits a peak at around 6 ºC, the position does not match the position observed in the crystalline COF structure composed of Tp and Bpy. TpTam-solid shows no peak at low angle region, meaning no formation of crystalline COF structure.

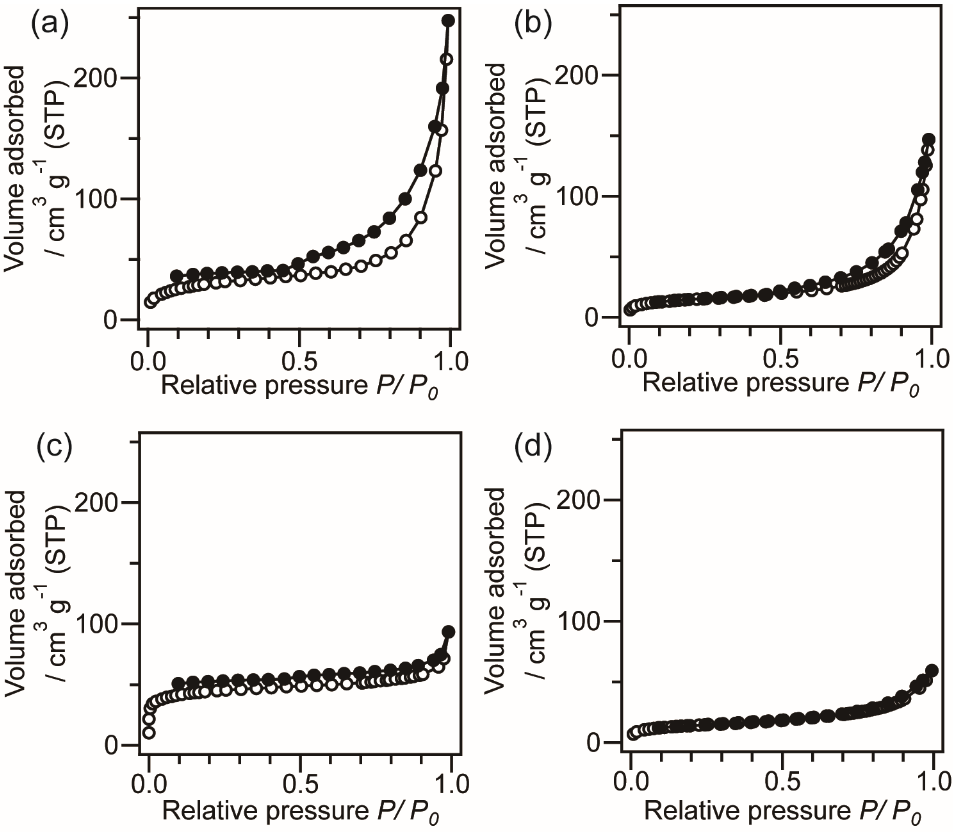

Figure S9. N_2 adsorption/desorption isotherms of (a) TpPa-solid, (b) TpBpy-solid, (c) TpTam-solid, and (d) TpDq-solid.

Comment for Figure S9: The N_2 adsorption isotherms of TpPa-solid, TpBpy-solid, and TpDq-solid show no significant uptake in the low-pressure region, suggesting the absence of micropores. In contrast, TpTam-solid exhibits a noticeable uptake at low relative pressure, indicating the presence of microporosity.

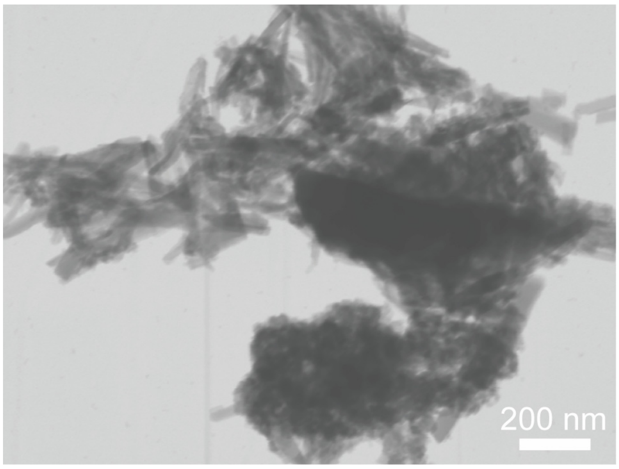

Figure S10. A degraded part in the STEM image of TpBpy-$KNiF_3$ after the N_2 adsorption/desorption measurement.

Comment for Figure S10: The SEM image of TpBpy-$KNiF_3$ after N_2 adsorption/desorption measurements reveals partial structural degradation, likely caused by the pre-heating process required for the measurement.

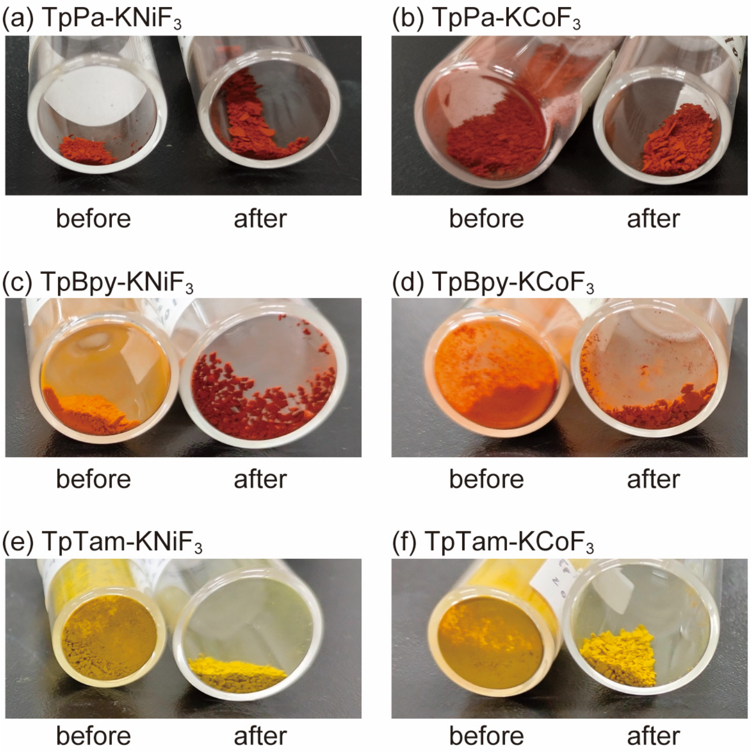

Figure S11. Appearance of the porous polymers before and after the N_2 adsorption/desorption measurements.

Comment for Figure S11: The sample colors of the TpPa and TpTam series remain unchanged after N_2 adsorption/desorption measurements. In contrast, the TpBpy series exhibit a pronounced color change following the measurement, suggesting that the degree of polymerization is altered by the pre-heating process required for N_2 adsorption/desorption analysis.

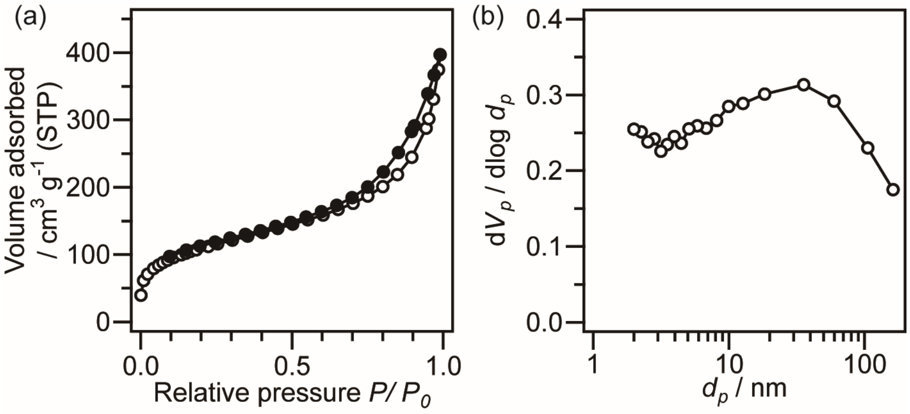

Figure S12. (a) N_2 adsorption/desorption measurement and (b) BJH pore size distribution of TpBpy-$KNiF_3$ heated at 80 ºC before the removal of $KNiF_3$.

Comment for Figure S12: TpBpy-$KNiF_3$ was subjected to heat treatment prior to the removal of the $KNiF_3$ template, followed by water washing. The resulting material exhibits a BET specific surface area of 396 m^2 g^{-1}. This high surface area can be attributed to the formation of a microporous crystalline structure. On the other hand, the BJH pore size distribution displays a broad peak in the mesoporous range (10–100 nm), suggesting partial structural degradation of mesostructures during the heat treatment process.

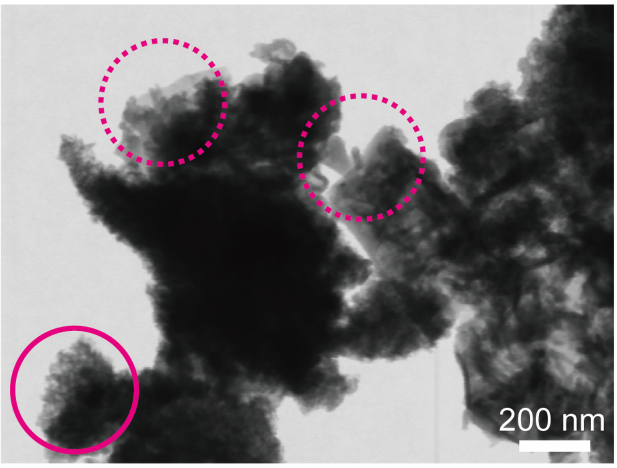

Figure S13. STEM image of Heated-TpBpy-$KNiF_3$. The dash-lined and solid-lined circle shows the crystallized parts and porous parts, respectively.

Comment for Figure S13: TpBpy-$KNiF_3$ was subjected to heat treatment prior to the removal of the $KNiF_3$ template, followed by water washing. The SEM image of the resulting material reveals predominantly non-porous regions, indicating crystallization during the heat treatment, although some porous domains are partially retained.

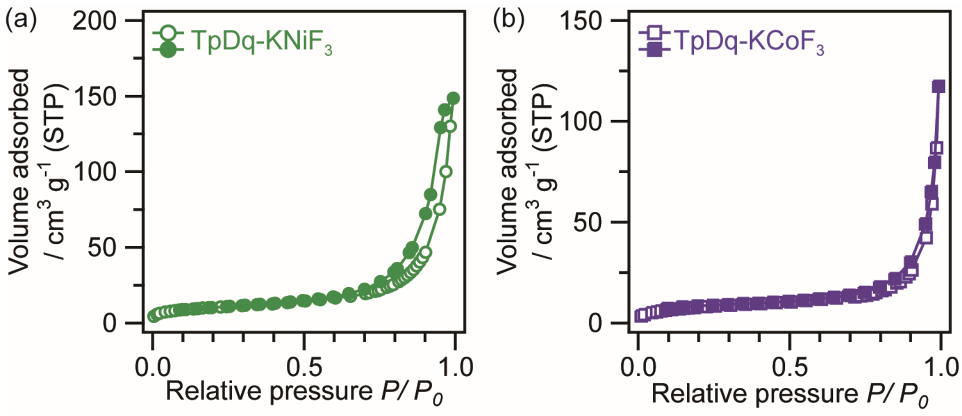

Figure S14. N_2 adsorption/desorption measurement of (a) TpDq-$KNiF_3$ and (b) TpDq-$KCoF_3$.

Comment for Figure S14: The N_2 adsorption isotherms of TpDq-$KNiF_3$ and TpDq-$KCoF_3$ exhibit no significant uptake, indicating the absence of well-defined porous structures.

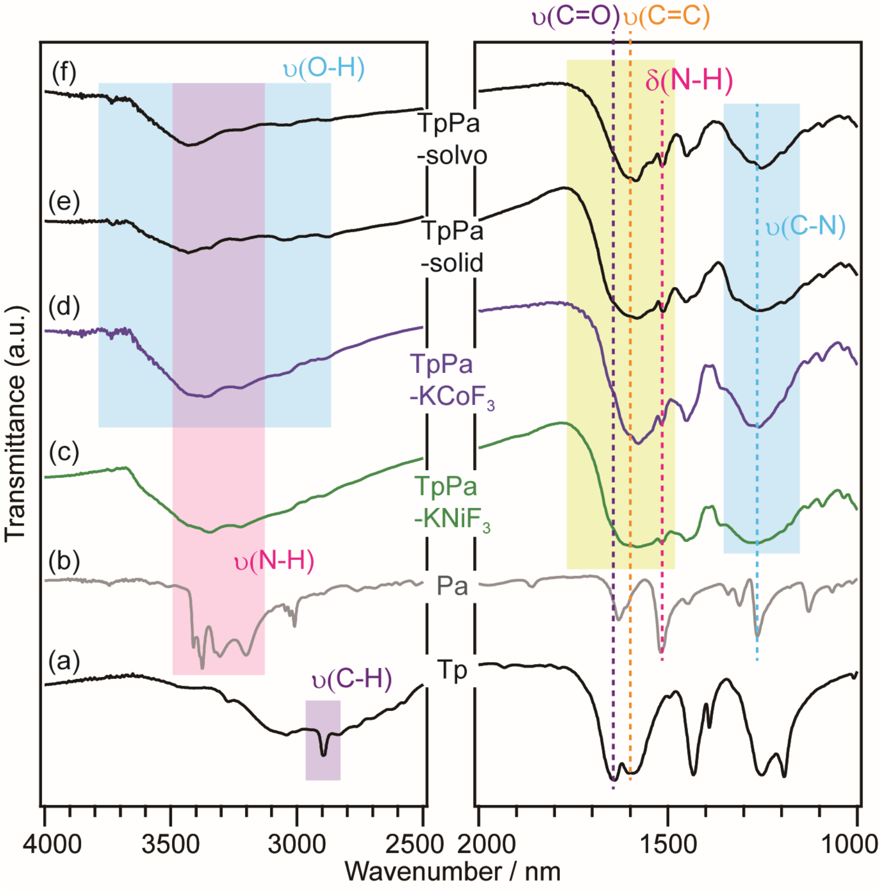

Figure S15. IR spectra of (a) Tp, (b) Pa, (c) TpPa-$KNiF_3$, (d) TpPa-$KCoF_3$, (e) TpPa-solid, and (f) TpPa-solvo.

Comment for Figure S15: The IR spectra of the TpPa series show a significant decrease in the intensity of bands associated with N–H and C–H vibrations, indicating the progression of the Schiff reaction between Tp and Pa.

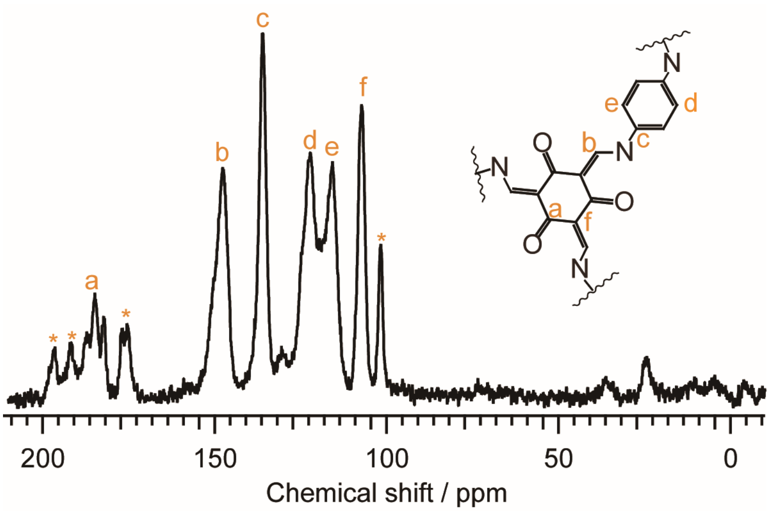

Figure S16. ^{13}C CP/MAS NMR spectrum of TpPa-solvo. The asterisks mark the peaks attributed to the unreacted Tp molecules.

Comment for Figure S16: The ^{13}C CP/MAS NMR spectrum of TpPa-solvo confirms the progress of the Schiff reaction between Tp and Pa. The presence of sharp peaks reflects the crystalline nature of TpPa. In contrast, the spectra of TpPa-$KNiF_3$ and TpPa-$KCoF_3$ exhibit broad peaks, indicative of their amorphous frameworks and lack of long-range order.

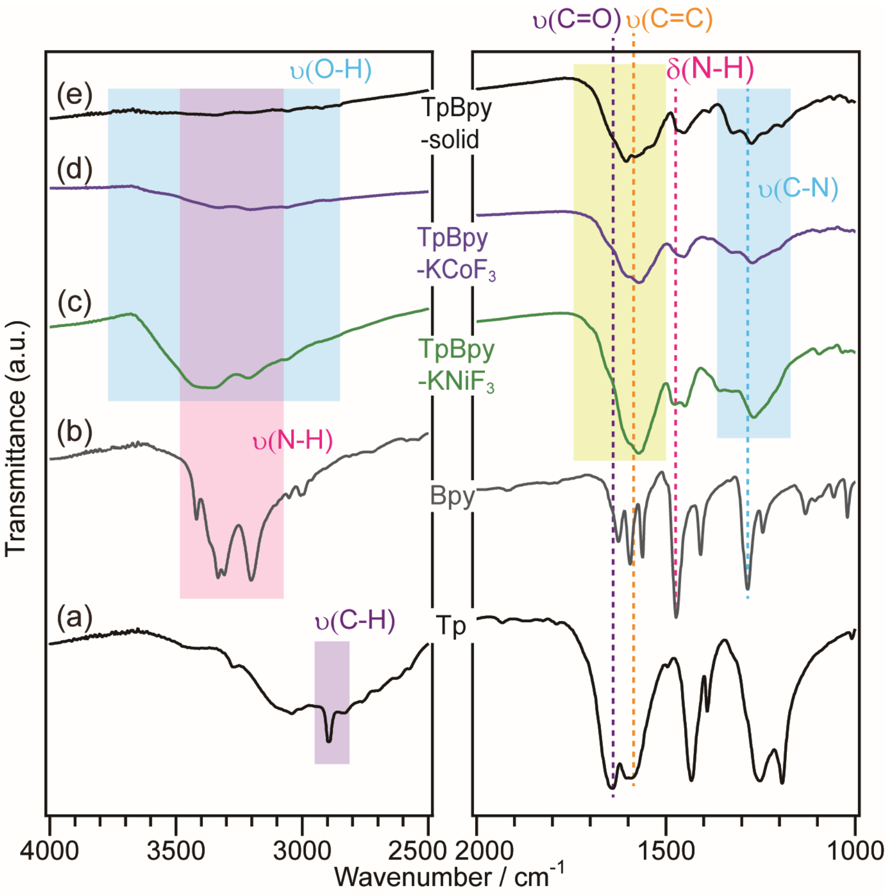

Figure S17. IR spectra of (a) Tp, (b) Bpy, (c) TpBpy-$KNiF_3$, (d) TpBpy-$KCoF_3$, and (e) TpBpy-solid.

Comment for Figure S17: The IR spectra of the TpBpy series show a significant decrease in the intensity of bands associated with N–H and C–H vibrations, indicating the progression of the Schiff reaction between Tp and Bpy.

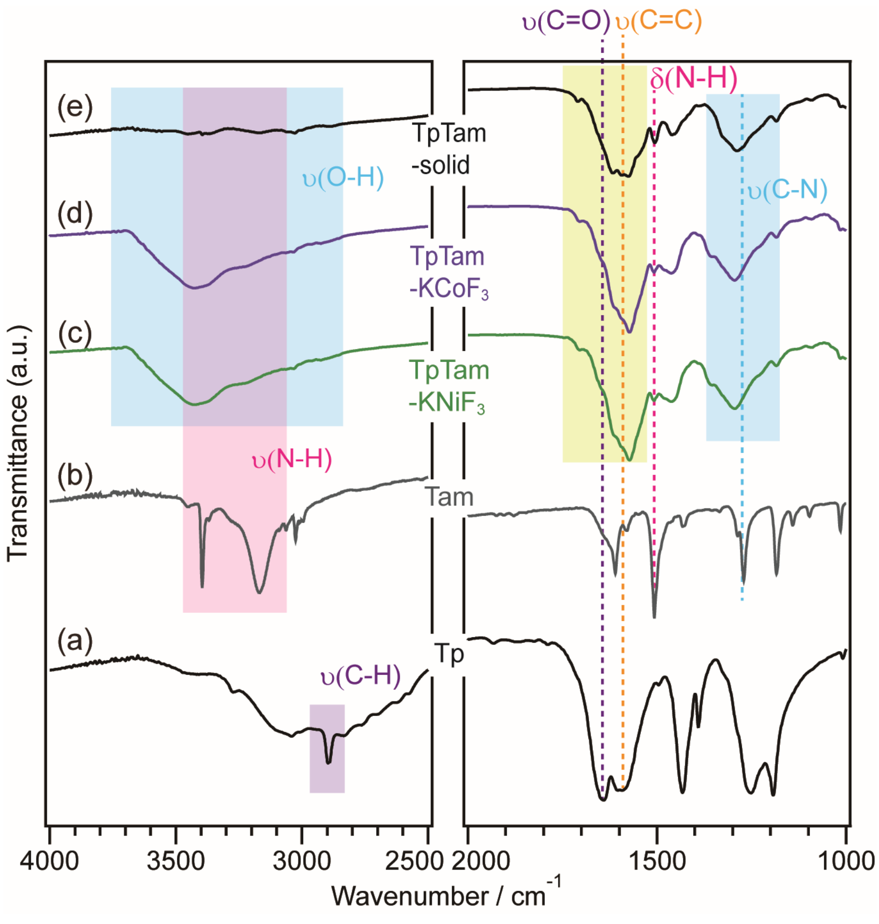

Figure S18. IR spectra of (a) Tp, (b) Tam, (c) TpTam-$KNiF_3$, (d) TpTam-$KCoF_3$, and (e) TpTam-solid.

Comment for Figure S18: The IR spectra of the TpTam series show a significant decrease in the intensity of bands associated with N–H and C–H vibrations, indicating the progression of the Schiff reaction between Tp and Tam.

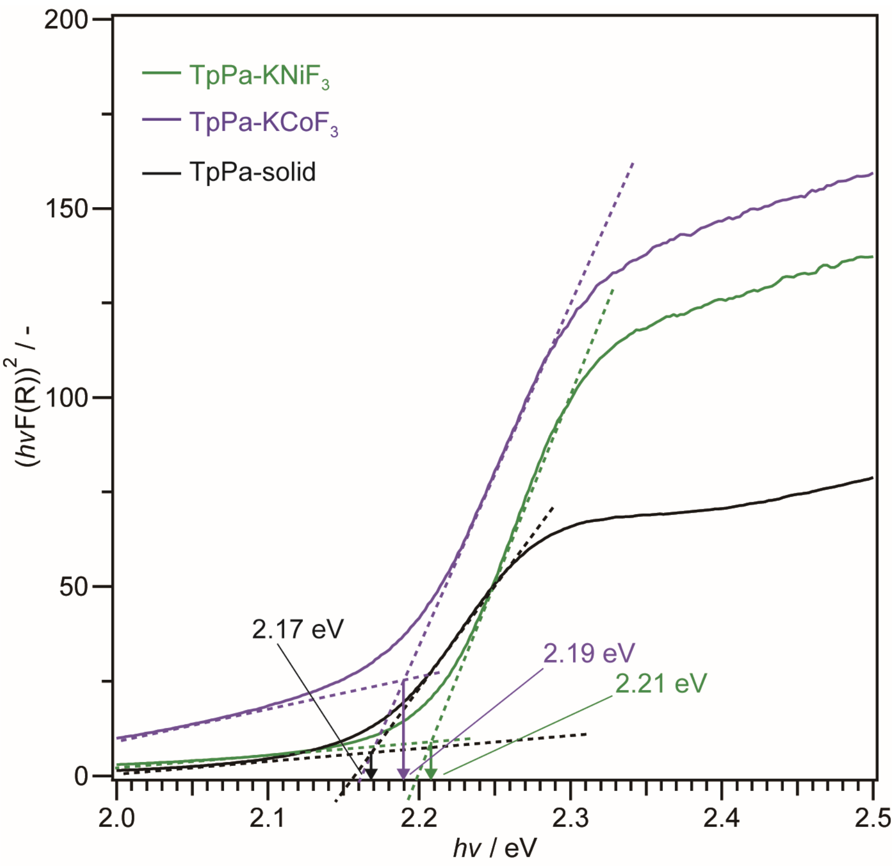

Figure S19. Tauc plots of TpPa-KNiF$_3$, TpPa-KCoF$_3$, and TpPa-solid.

Comment for Figure S19: The Tauc plots of TpPa-KNiF$_3$, TpPa-KCoF$_3$, and TpPa-solid were used to estimate their band gaps, applying the equation for direct electronic transitions. The resulting band gaps show slight variations, which are likely attributed to differences in the degree of polymerization among the samples.

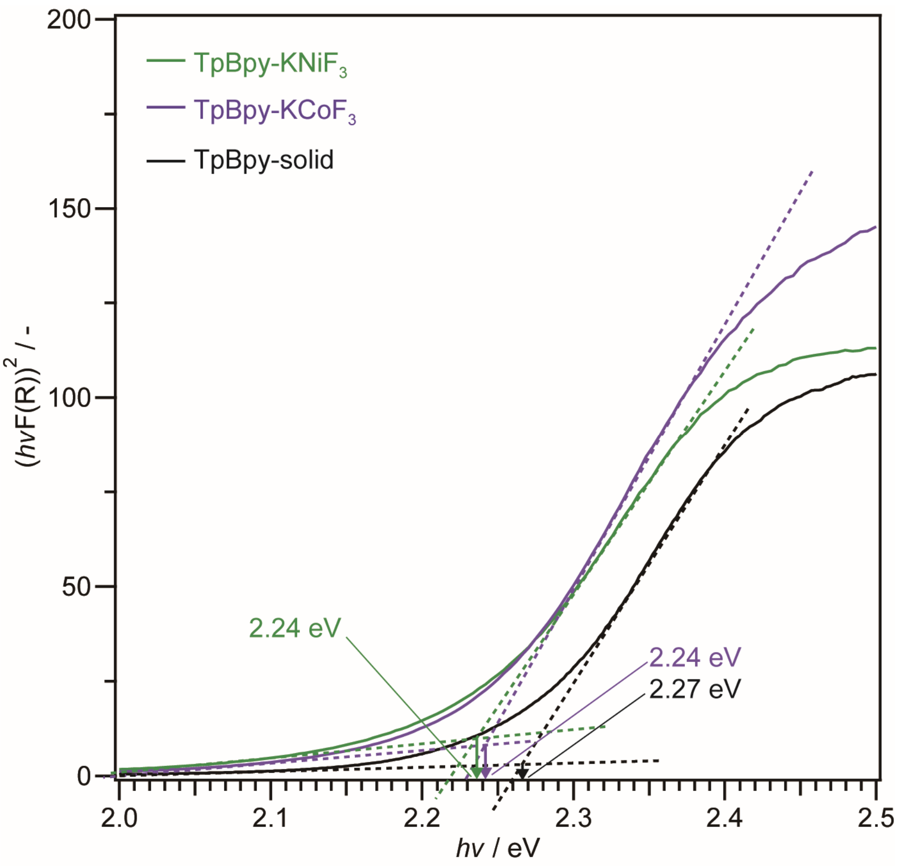

Figure S20. Tauc plots of TpBpy-$KNiF_3$, TpBpy-$KCoF_3$, and TpBpy-solid.

Comment for Figure S20: The Tauc plots of TpBpy-$KNiF_3$, TpBpy-$KCoF_3$, and TpBpy-solid were used to estimate their band gaps, applying the equation for direct electronic transitions. The resulting band gaps show slight variations, which are likely attributed to differences in the degree of polymerization among the samples.

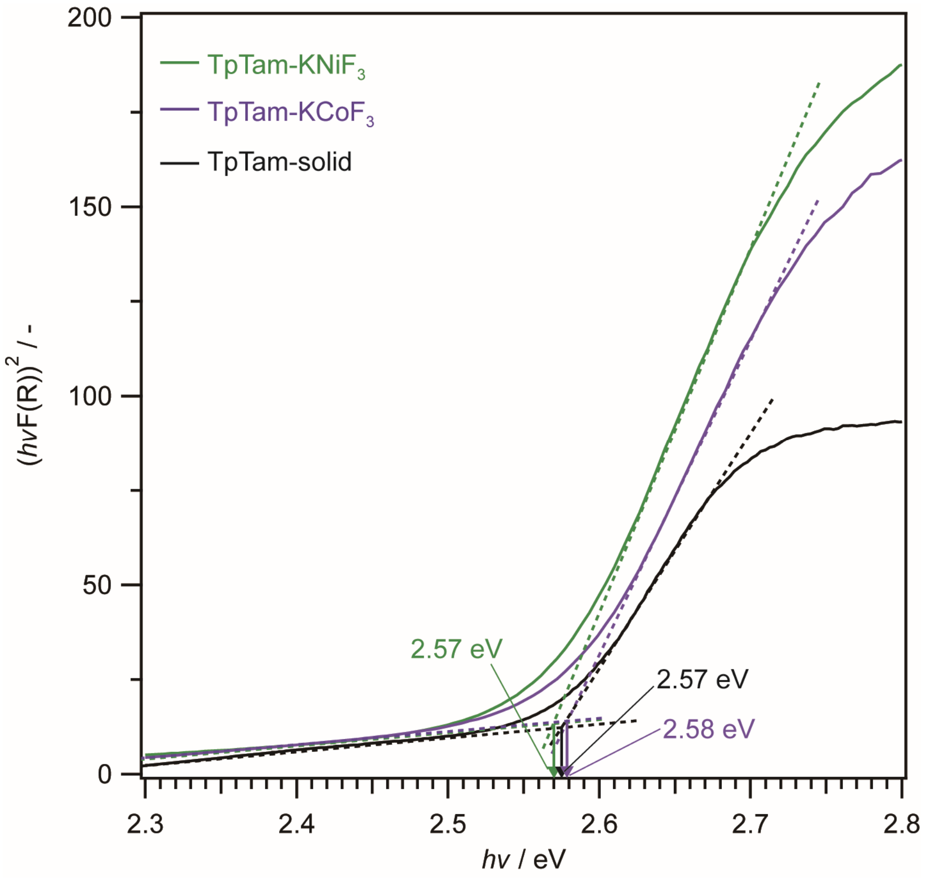

Figure S21. Tauc plots of TpTam-KNiF$_3$, TpTam-KCoF$_3$, and TpTam-solid.

Comment for Figure S21: The Tauc plots of TpTam-KNiF$_3$, TpTam-KCoF$_3$, and TpTam-solid were used to estimate their band gaps, applying the equation for direct electronic transitions. The resulting band gaps show slight variations, which are likely attributed to differences in the degree of polymerization among the samples.

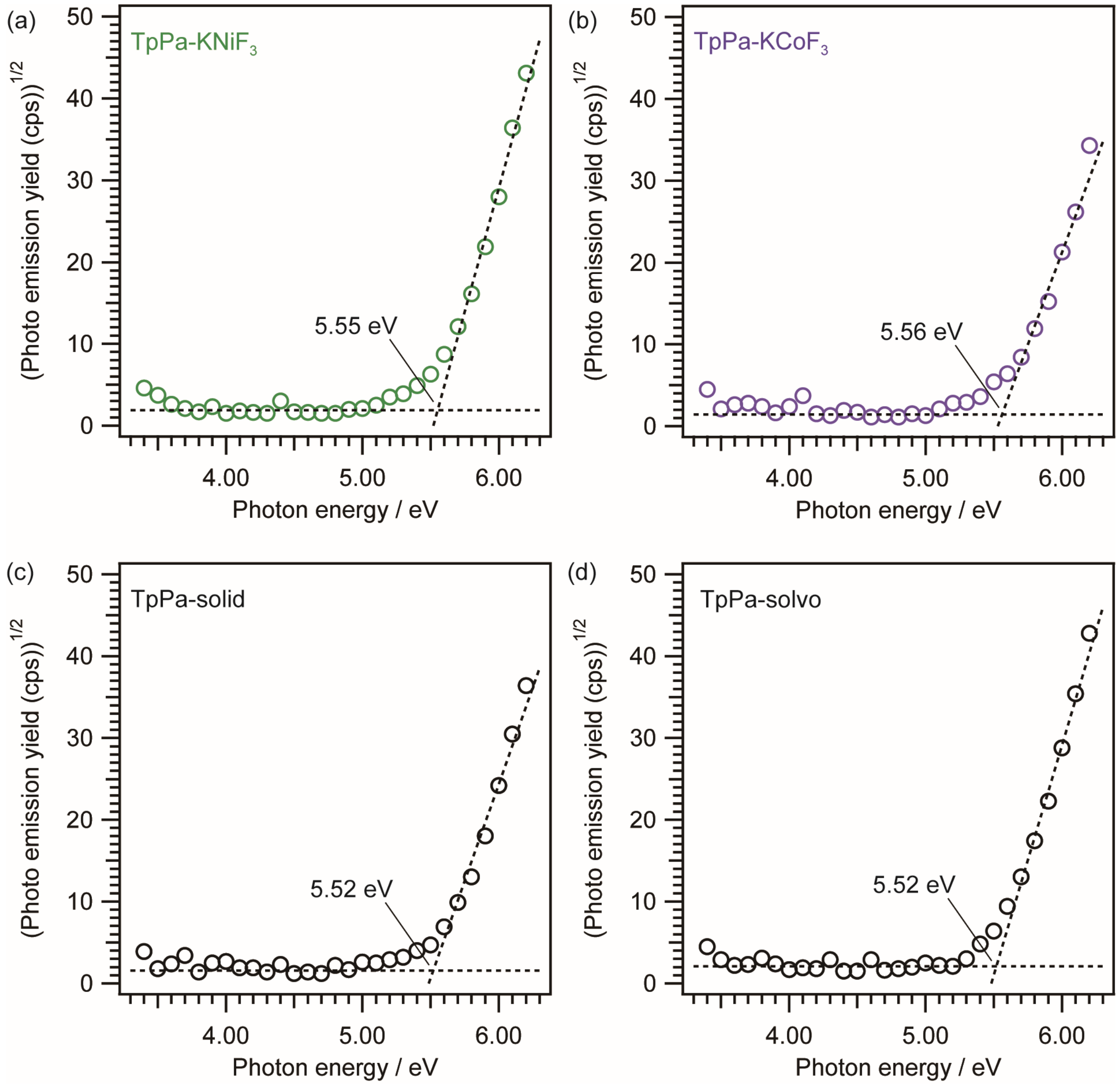

Figure S22. PYSA spectra of (a) TpPa-$KNiF_3$, (b) TpPa-$KCoF_3$, (c) TpPa-solid, and (d) TpPa-solvo.

Comment for Figure S22: PYSA was used to determine the valence band maximum (VBM) relative to the vacuum level. The TpPa series exhibit similar VBM positions, indicating comparable electronic structures across the samples.

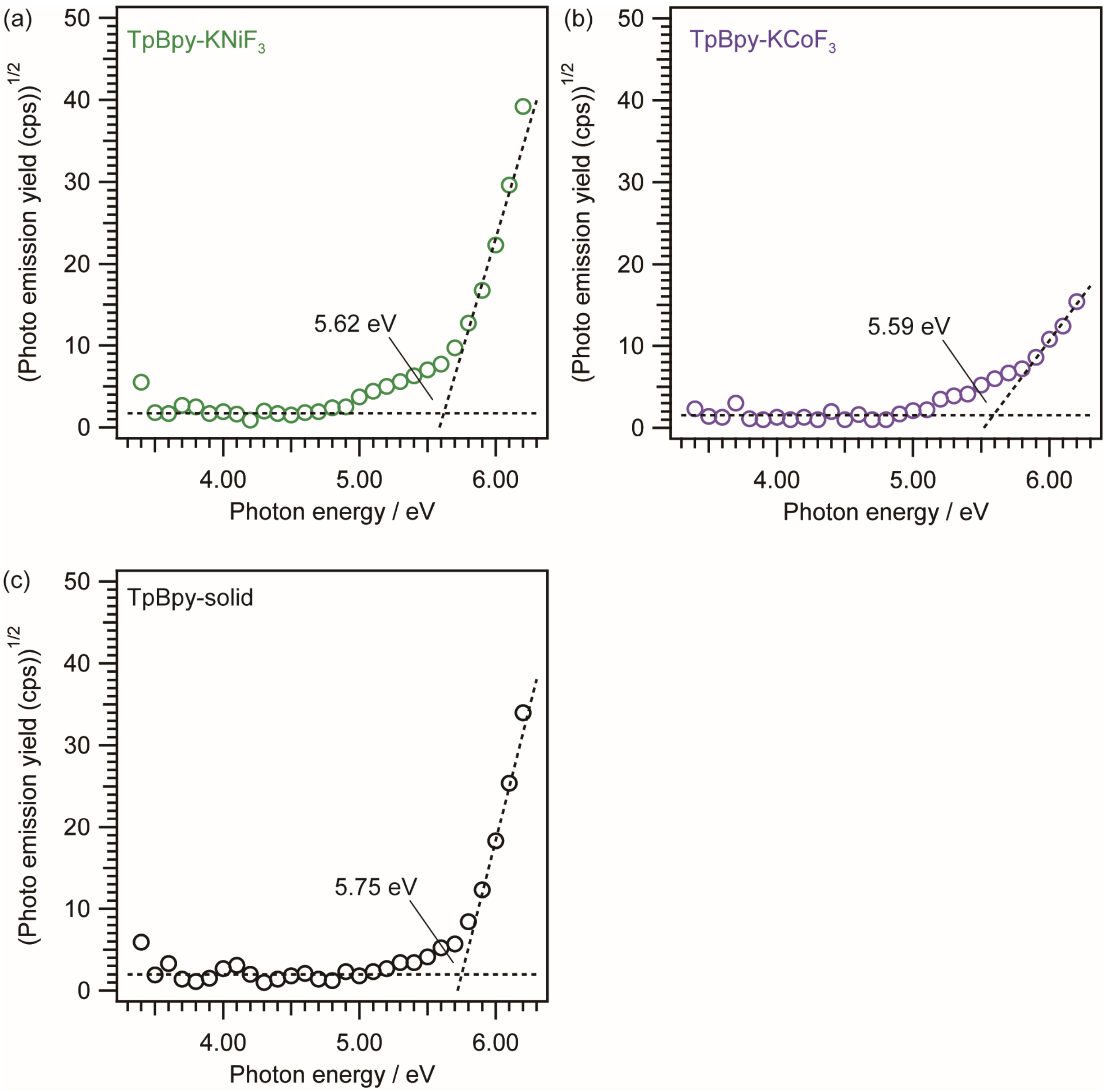

Figure S23. PYSA spectra of (a) TpBpy-$KNiF_3$, (b) TpBpy-$KCoF_3$, and (c) TpBpy-solid.

Comment for Figure S23: PYSA was employed to determine the valence band maximum (VBM) relative to the vacuum level. TpBpy-solid exhibits a slightly deeper VBM compared to the porous samples, likely due to differences in the degree of polymerization, which can influence the electronic structure.

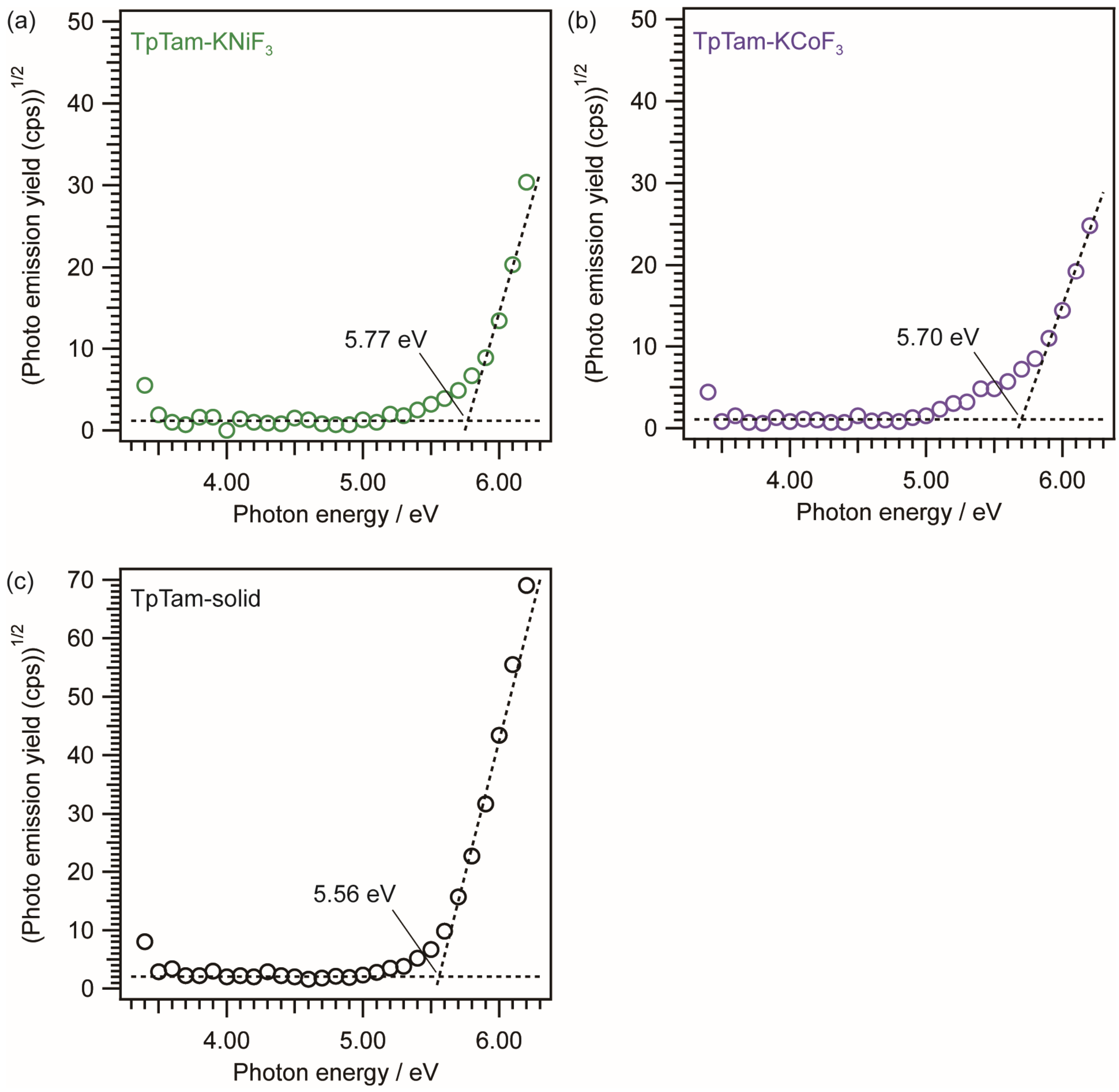

Figure S24. PYSA spectra of (a) TpTam-$KNiF_3$, (b) TpTam-$KCoF_3$, and (c) TpTam-solid.

Comment for Figure S24: PYSA was employed to determine the valence band maximum (VBM) relative to the vacuum level. TpTam-solid exhibits a slightly deeper VBM compared to the porous samples, likely due to differences in the degree of polymerization, which can influence the electronic structure.

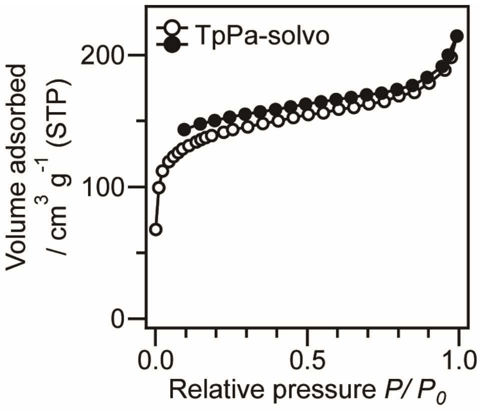

Figure S25. N_2 adsorption/desorption measurement of TpPa-solvo.

Comment for Figure S25: The BET specific surface area is measured to be 524 m^2 g^{-1}, which can be attributed to the presence of micropores arising from the crystalline COF structure.

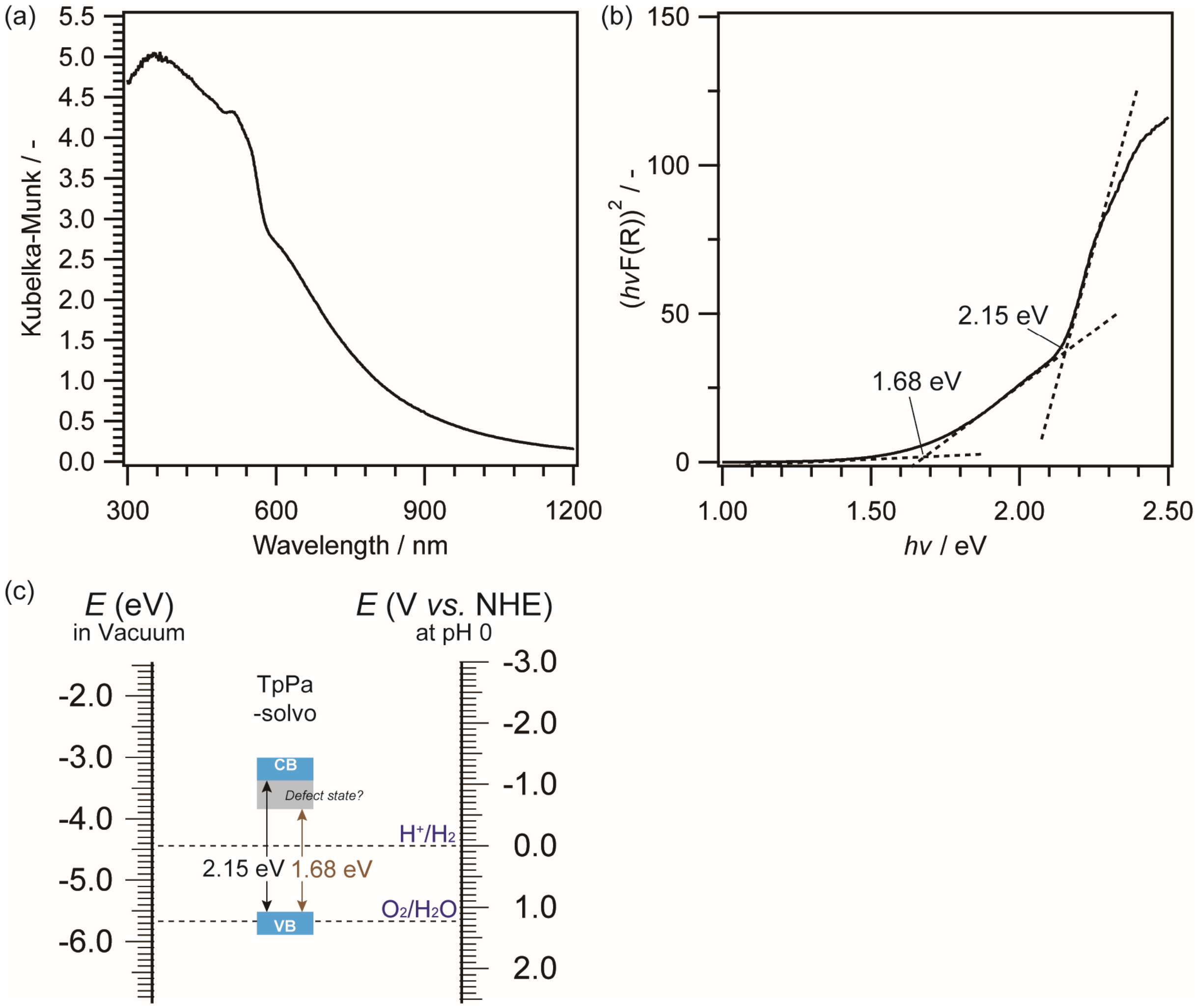

Figure S26. (a) UV-vis spectrum, (b) Tauc plot, and (c) Proposed band structure of TpPa-solvo.

Comment for Figure S26: The Tauc plot of TpPa-solid reveals two absorption components. As shown in Figure S23c, this suggests the presence of defect states located slightly below the conduction band minimum.

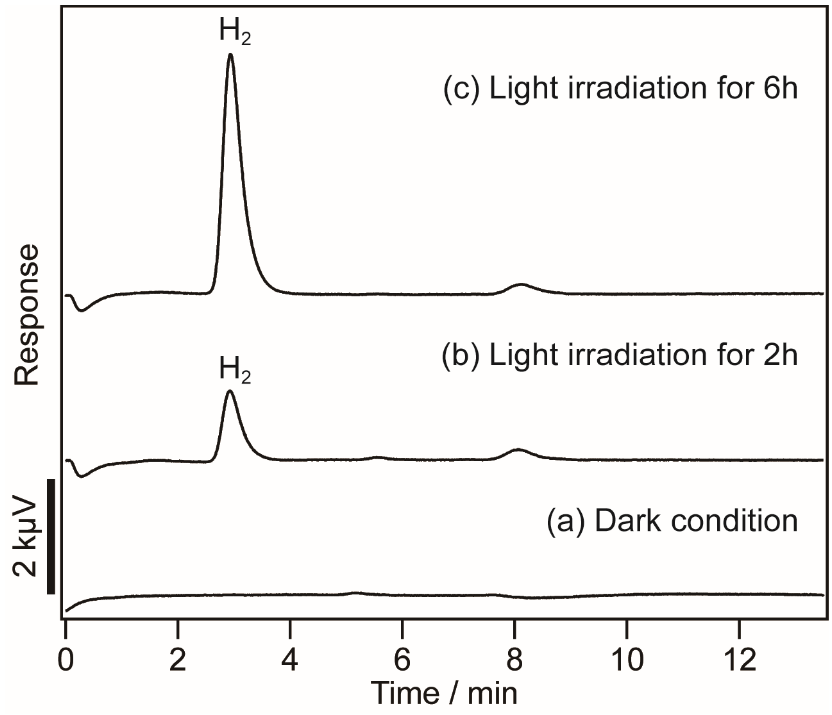

Figure S27. Gas chromatography profile of photocatalytic hydrogen evolution evaluation for TpPa-$KCoF_3$ (a) under dark condition and under light irradiation for (b) 2 h and (c) 6 h.

Comment for Figure S27: Under dark conditions with TpPa-$KCoF_3$, no hydrogen evolution is detected (a). In contrast, under light irradiation, hydrogen is generated, and the amount steadily increases with irradiation time (b and c).

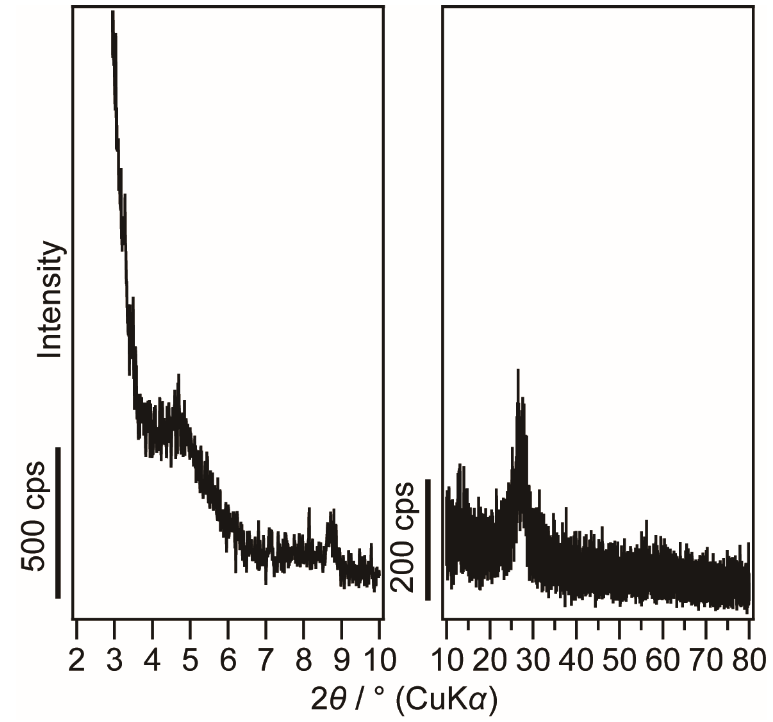

Figure S28. XRD pattern of TpPa-solvo.

Comment for Figure S28: The XRD pattern of TpPa-solvo confirms its crystalline structure, which is consistent with its observed microporous nature.

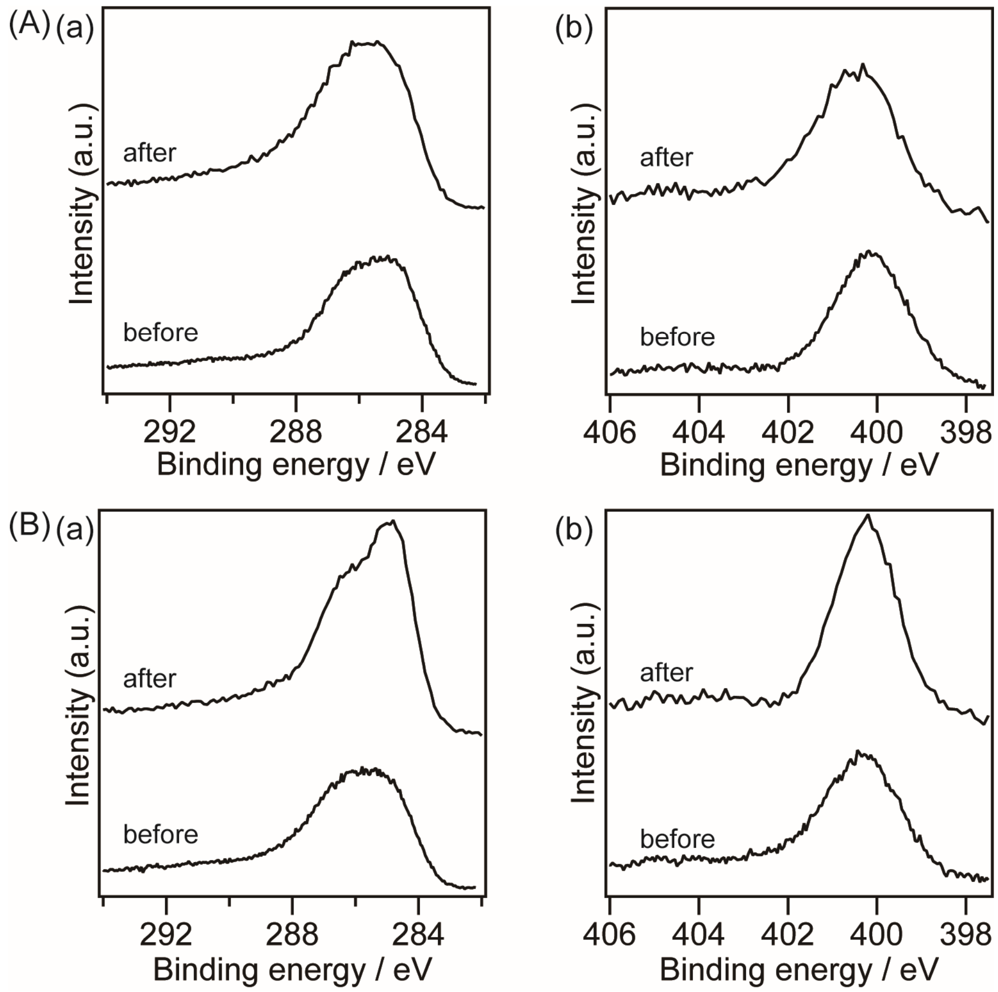

Figure S29. XPS (a) C 1s and (b) N 1s spectra of (A) TpPa-$KNiF_3$ and (B) TpPa-$KCoF_3$ before and after photocatalytic hydrogen evolution reactions.

Comment for Figure S29: The C 1s spectrum of TpPa-$KCoF_3$ after photocatalysis (Ba) shows a new peak at 284.8 eV, likely corresponding to C–C bonding from ascorbic acid and/or its decomposition products. In the spectrum of TpPa-$KNiF_3$ (Aa), the broad nature of the signal may include contributions from ascorbic acid and/or its derivatives. In the N 1s spectra of TpPa-$KNiF_3$ and TpPa-$KCoF_3$ (Ab and Bb), no significant changes are observed before and after the reaction.

Table S1. BET surface areas of the TpDq series calculated from N_2 adsorption isotherms.

Sample names	BET surface area / m^2 g^{-1}
TpDq-solid	51
TpDq-$KNiF_3$	38
TpDq-$KCoF_3$	30

Table S2. Relative compositional ratios of the TpPa-series samples based on the XPS results.

Sample names	C	N	O	Ni	Co
TpPa-$KNiF_3$	36	5.7	11.0	1.3	-
TpPa-$KCoF_3$	36	5.5	11.5	-	1.4
TpPa-solid	36	5.7	9.3	-	-
TpPa-solid	36	6.9	5.8	-	-
The ideal crystal structure of TpPa	36	6	6	-	-

*The values were calculated by normalizing the carbon content to a fixed value of 36 atoms.
